# Neuroinflammation and the Kynurenine Pathway in CNS Disease: Molecular Mechanisms and Therapeutic Implications

**DOI:** 10.3390/cells10061548

**Published:** 2021-06-19

**Authors:** Mustafa N. Mithaiwala, Danielle Santana-Coelho, Grace A. Porter, Jason C. O’Connor

**Affiliations:** 1 Integrated Biomedical Sciences Program, Graduate School of Biomedical Sciences, UT Health San Antonio, San Antonio, TX 78229, USA; mithaiwala@livemail.uthscsa.edu (M.N.M.); coelhod@livemail.uthscsa.edu (D.S.-C.); porterg3@livemail.uthscsa.edu (G.A.P.); 2 Department of Pharmacology, Long School of Medicine, UT Health San Antonio, Mail Code 8864, San Antonio, TX 78229, USA; 3 Department of Research, Audie L. Murphy VA Hospital, South Texas Veterans Heath System, San Antonio, TX 78229, USA

**Keywords:** affective disorders, depression, kynurenine pathway, microglia, neuroinflammation, neurodegeneration, therapeutic strategies

## Abstract

Diseases of the central nervous system (CNS) remain a significant health, social and economic problem around the globe. The development of therapeutic strategies for CNS conditions has suffered due to a poor understanding of the underlying pathologies that manifest them. Understanding common etiological origins at the cellular and molecular level is essential to enhance the development of efficacious and targeted treatment options. Over the years, neuroinflammation has been posited as a common link between multiple neurological, neurodegenerative and neuropsychiatric disorders. Processes that precipitate neuroinflammatory conditions including genetics, infections, physical injury and psychosocial factors, like stress and trauma, closely link dysregulation in kynurenine pathway (KP) of tryptophan metabolism as a possible pathophysiological factor that ‘fuel the fire’ in CNS diseases. In this study, we aim to review emerging evidence that provide mechanistic insights between different CNS disorders, neuroinflammation and the KP. We provide a thorough overview of the different branches of the KP pertinent to CNS disease pathology that have therapeutic implications for the development of selected and efficacious treatment strategies.

## Highlights

- Disturbances in KP metabolism and its regulation affect CNS disease progression and associated pathology with notable changes in KP metabolite levels, enzyme function and gene induction.
- KP of tryptophan metabolism is ubiquitous in eukaryotic cells and regulates several key biological systems including oxidative stress, energy metabolism, immune function, gut-microbiota functions and neurotransmitter functions.
- Aging, genetic and environmental risk factors ensue a feedforward loop between neuroinflammation and KP metabolic imbalance during CNS disease.
- Broadly targeting neuroinflammation in CNS disorders with currently available anti-inflammatory pharmacotherapy is inefficacious for a lack of specificity.
- Therapeutic targeting of KP for CNS diseases requires a better understanding of KP metabolite functions, cellular and molecular events affected by KP and neuroinflammation in effector cells like microglia and astrocytes.
- In this review, we describe and discuss evidence from the field that elaborates on KP metabolite production and function, alterations in KP metabolic imbalance arising during CNS diseases and potential targets tested in pre-clinical or clinical stages.

## 1. Introduction

Central nervous system disorders are the second-leading cause of death globally (~17%), and they pose a major healthcare problem affecting over 250 million people worldwide when accounting for disability adjusted life years (DALYs) [1]. In the United States alone, Alzheimer’s disease (AD) and other dementias, Parkinson’s disease (PD), chronic lower back pain, stroke, traumatic brain injury (TBI), migraines, epilepsy, multiple sclerosis (MS) and spinal cord injury are the most commonly reported neurological disorders that cumulatively amount to a significant burden on the healthcare expenditure (approximately USD 0.8 trillion) and caregivers. Among these, AD and other dementias affect over 7.5 million people in the US with the average per person cost of care estimated at USD 46,000 and over USD 240 billion in total expenditure [2]. The US Census Bureau predicts that by 2050 over 88.5 million people will be 65 years and older, or one out of every four people, indicating an increasing aging populous that will be at elevated risk for disease.

Neuropsychiatric and neurodevelopmental disorders are not among the leading cause of deaths in the US; however, the disease burden and prevalence of these mental health conditions is both alarming and overwhelming. According to the National Institute of Mental Health (NIMH), neurological disorders in conjunction with neuropsychiatric disorders are the leading contributors to DALYs and years lived with disability (YLD) ahead of cardiovascular and circulatory diseases among the American population. However, globally stroke remains the single largest neurological contributor to DALYs. Globally, the prevalence of mental health disorders including substance abuse is 11–18% or 1 in every 6–7 people [1]. Over 264 million people in the world suffer from depression alone while over 275 million people live with at least one type of anxiety disorder. As of 2017, an estimated one in five US adults or 14% of the general population lived with a mental illness. The most prevalent of mental health disorders are anxiety disorders with over 40 million or 18.1% of the population affected by them (Anxiety and Depression Association of America). There are an estimated 17.4 million adults or 7.1% of the US population who have suffered at least one major depressive disorder (MDD), which is less prevalent than anxiety disorders but remains the dominant contributor to US DALYs affected by mental illnesses (National Institute of Mental Health). Around the world, mental health problems cause 7% of all global burden of disease and account for 19% years lived with disability. A myriad of factors have led to the current scenario that includes cuts to spending on neurological and mental health research, inefficient and inadequate government spending on healthcare, lack of a holistic understanding of the human brain, mysterious etiological origins of CNS diseases and a dearth of appropriate tools and disease models to study the brain and its disorders. Frustratingly, where there have been remarkable achievements in other diseases and associated therapeutics, candidate drugs and strategies to treat CNS disorders have met with limited success in the clinic. There is an urgent and unmet demand for targeted therapeutics that are able to mitigate CNS conditions, which relies on a better understanding of the pathogenic mechanisms that underlie the fundamental origins of such disorders. Over the past two decades, a burgeoning amount of literature has implicated the role of innate immune response and closely associated neuroinflammation to be a critical risk and pathological factor of CNS disorders. Neuroinflammation is prevalent in multiple brain disorders including AD, TBI, stroke, anxiety and mood disorders, neurodevelopmental disorders [3,4,5]. There is remarkable similarity in biochemical observations, cellular and molecular changes and outcomes of behavioral experiments between clinical settings and preclinical models of neuroinflammation that support this notion. Importantly, there exists an intricate relationship between neuroinflammation and the innate immune response, designed to repair and protect the organism, but dysregulation of the same processes due to numerous factors can be detrimental to the organism and its survival.

Alterations in metabolic pathways are an important consequence that arise due to inflammatory process. One such pathway that has received considerable attention in the recent past has been the kynurenine pathway (KP) of tryptophan metabolism, which is most well-known for the de novo synthesis of nicotinamide adenine dinucleotide (NAD). NAD is present in all eukaryotic cells. It is the ultimate breakdown product of oxidative kynurenine metabolism critically involved in redox reactions of energy metabolism along the mitochondrial respiratory chain, DNA repair and transcriptional regulation and as a novel neurotransmitter [6]. In addition to NAD, several other metabolic products of the KP exist that exert unique biological actions, and will be described herein. Importantly, studies from both the laboratory and the clinic have reported alterations in KP metabolism and fluctuations in the level of KP metabolites in the context of CNS disease. As appreciation that inflammation-induced changes in KP metabolism may represent a convergent pathogenic target across a wide spectrum of CNS disease, understanding cellular and molecular mechanisms are necessary to develop novel therapeutic strategies.

## 2. Neuroinflammation

Neuroinflammation is the inflammation of the CNS that arises due to disease, brain injury, infection or stress, which involves the production and complex interplay of cytokines, chemokines, reactive oxygen species and second messengers. Numerous studies have reported the involvement of neuroinflammation in diseases such as AD, PD, stroke, TBI, mood disorders and autism spectrum disorders (ASD) [3,7]. In AD, a vicious loop between neuronal damage due to amyloid-β (Aβ) aggregation and neurofibrillary tangles, neuroinflammation and microglia activation exists that correlates well with the progression of disease and symptom severity with extensive damage found in cortical and basal forebrain areas [4,8]. Interestingly, there is evidence to suggest that microglia elimination in the five familial mutations (5xFAD) model of AD does not modulate Aβ pathology [9]. A follow up study by the same group published found that longer treatment with colony stimulating factor 1 receptor (CSF1R) antagonist which eliminates microglia for longer periods did indeed impair plaque formation and that microglia are key in plaque formation [10]. In addition, a recently published study also found that tau pathology is not affected by the presence of peripheral macrophages nor microglia elimination in young or old human Tau knock-in tau transgenic mice (hTau) [11]. Of note, the hTau model does not fully recapitulate the diseased AD brain and develops pathology on a different time course compared to other models like the 5xFAD model. Furthermore, recent studies have shown that age associated changes in microglial genes of humans and mice align well at younger ages; however, they do differ at older ages especially disease associated ones and have little overlap. It is attractive to speculate that this phenomenon could explain little effect of microglia elimination in hTau mice [12,13]. Clinical studies assaying inflammatory cytokine production, neuroimaging and post-mortem human brain tissue transcriptomic analysis have better predictive validity while assaying pathological hallmarks [14]. A meta-analysis conducted by Swardfager et al., found elevated levels of pro-inflammatory cytokines like interleukin-1 beta (IL-1β,) tumor necrosis factor-alpha (TNF-α), interleukin-6 (IL-6) and transforming growth factor-beta (TGF-β) in blood samples of AD patients and elevated levels of TGF-β in cerebrospinal fluid (CSF) of AD compared to healthy controls [15]. Although TGF-β is protective, higher circulating levels of IL-6 promote TGF-β signaling in microglia; the resident immune cells of the brain that prime microglia and increase their activation. Microglia activation is characterized by changes in both structure and function where they transition from highly ramified/branched surveilling cells to de-ramified, spheroidal cells that release pro-inflammatory cytokines and chemokines primed for clearing cellular and pathogenic debris [16]. Similarly, stroke, which is associated with major damage to the neurovasculature, induces a robust neuroinflammatory component that lasts several days to weeks. This period involves the activation of microglia cells around the infarct area, followed by infiltration of leukocytes, neutrophils and others inflammatory cells, increased production of reactive oxygen species (ROS), cytokines, chemokines and increased immune signaling mechanisms to provide damage control in a spatio-temporal manner [3]. However, the sustained microglial activation and presence of such neuroinflammatory processes in the infarct area affects the neighboring peri-infarct region to exacerbate excitotoxicity and upregulating inflammatory signaling mechanisms. In neurodegenerative and neurological diseases like AD, PD, stroke or epilepsy, glial cells (astrocytes and microglia) are the first line of cellular defense that respond to local and global insults. Activation of these cells engages and activates several mechanisms including aberrant cytokine and chemokine signaling, alterations in B-cell and T-cell response, caspase and complement activation and recruitment of peripheral macrophages, leukocytes and neutrophils in the brain. In turn, dysregulated neuroinflammation can result in altered metabolism, increased demyelination, neuronal apoptosis, neuronal autophagy and perturbed mitochondrial energetics that compromise the functioning of nerve cells ultimately causing their death and become pathological features observed in patients [17,18]. In addition, chronic inflammation induced experimentally in rodents can reduce rates of neurogenesis, cause dendritic atrophy of pyramidal neurons and alter density and stability of neuronal spines (synapses) [19,20]. The precise mechanism(s) by which neuroinflammation precipitates these diverse pathogenic processes in the CNS remain poorly understood, but KP metabolism has emerged as a putative point of convergence.

Affective disorders that include major depressive disorder (MDD), anxiety disorders and schizophrenia usually do not show overt neuronal loss typically observed in neurological and neurodegenerative diseases; however, extensive literature suggests that psychiatric illnesses also have a prominent neuroimmune/innate immune signature that positively correlates with clinical symptoms. These include an increase in the presence of pro-inflammatory markers like TNF-α and IL-6 in both the periphery and CSF of patients accompanied with an increase in acute phase proteins like C-reactive protein (CRP) and an upregulated innate immune response [21,22]. From an evolutionary perspective, these signals allow efficient management of threats and aversive stimuli to regulate mood and reward processing, but risk factors, like abnormal gene processing, persistent inflammatory signals associated with a host of peripheral diseases, and chronic stress can turn this in to a maladaptive immune response that becomes pathogenic. Observations from a clinical study by Capuron et al., where patients with malignant melanoma being treated with interferon-α (IFN-α) noted that immunotherapy resulted in marked changes in mood of patients similar to symptoms seen in patients suffering from MDD and anxiety disorders [23]. Moreover, in a subset of these patients, co-treatment with the classical anti-depressant paroxetine belonging to the selective serotonin reuptake inhibitors (SSRIs) class did not improve symptoms of anxiety and depression induced by cytokine therapy. The authors concluded that the observed effects on mood after treatment with the inflammatory cytokine were not a result of sickness behavior but other mechanisms were at play that would explain the affective changes observed in such patients [23].

One significant finding that could provide a plausible explanation was a marked decrease in serum tryptophan levels. Remarkably, a wide range of studies have reported increased tryptophan metabolism along the KP in patients suffering neurologic, neurodegenerative, neuropsychiatric and neurodevelopmental disease. Experimentally, direct administration of endotoxin (LPS), a pathogen associated molecular pattern (PAMP) found in the cell wall of Gram-negative bacteria, induces anxiety and depressive like behaviors in healthy human volunteers as well as in mouse models. Targeted genetic deletion of indoleamine-2,3-dioxygenase (IDO) or pretreatment of mice with its inhibitor, 1-methyltryptophan (1-MT), protected mice from the anxiogenic, depressogenic, and cognitive impairing effects produced by administration of LPS [24,25]. Furthermore, LPS and other infectious agents including viruses upregulate the production of inflammatory cytokines by engaging Toll-like receptors (TLRs) which, in turn, induce enzymes along the KP of tryptophan metabolism both directly and indirectly via proinflammatory cytokines.

Therapeutic attempts to broadly targeting inflammation/neuroinflammation in CNS disease have yielded largely disappointing results. The INTREPAD trial evaluated the safety and efficacy of low-dose Naproxen, a non-steroidal anti-inflammatory drug (NSAID) for preventing the progression of disease in presymptomatic AD among cognitively intact persons at risk. However, not only did it fail to reduce the progression of presymptomatic AD but also found an increase in the frequency of adverse effects associated with the treatment of Naproxen 220 mg twice daily [26]. Similarly, other clinical trials that include administration of drugs belonging to the NSAID class, rofecoxib, celecoxib, diclofenac, indomethacin and the nuclear factor kappa light chain enhancer of activated B cells (NF-κB) inhibitor Etanercept failed to show significant beneficial effects on AD progression. Moreover, the side effects on cardiovascular function and gastrointestinal function outweighed any marginal beneficial effect observed from such studies [27,28]. Likewise, broad strategies such as targeting leukocyte infiltration, interleukin-1 receptor (IL-1R) antagonism and immunomodulation of T-regulatory cells have failed to improve stroke outcomes in preclinical and clinical testing [29]. For affective conditions like MDD, treatment with IL-6 targeting antibodies sirukumab and tocilizumab, NSAIDs like acetyl salicylic acid (ASA) and celecoxib, N-Acetylcysteine that reduces oxidative stress have met with limited success while treatment with TNF-α antagonist infliximab shows beneficial effects in only a subset of patients with higher inflammatory markers profile [30]. Yet, in the same diseases or disease models, more targeted treatments focused on discrete or downstream inflammatory processes and effector cells have shown more promise. For example, in a double-blind randomized clinical trial with treatment resistant depression patients, found augmentation of minocycline (a tetracycline antibacterial) to anti-depressants improved behavioral symptoms in patients with low grade inflammation [31]. A meta-analysis also found minocycline to have significant anti-depressant activity and several clinical trials aimed to improve depression outcomes and improve treatment response scores in mood disorders with minocycline are currently underway [32,33]. LPS induced depressive like alterations are reversed by treatment with ketamine that positively correlated with reduction in quinolinic acid (QA) production from microglia [34]. A novel antagonist of purinergic type 2 (P2X7) receptors, which are critical for microglial activation, was efficacious in mitigating depressive-like behaviors induced by peripheral inflammation or chronic stress in rodents [35]. Furthermore, targeting microglia activation using minocycline has shown utility in preclinical models of cerebral ischemia and additional clinical trials are required to evaluate translational benefits in improving stroke outcomes [36]. Interestingly, targeting the KP oxidative branch to block downstream metabolite production by administration of kynurenic acid or inhibiting the KMO enzyme in rodent models of stroke have yielded positive results [37]. However, these strategies have not been tested in clinical trials. For neurodegenerative diseases like AD and PD, minocycline treatment failed to slow cognitive decline or significantly improve disease progression in clinical trials [38,39]. However, microglia ablation in the 5xFAD model of AD using the CSF-1R inhibitor-PLX5622 reduced plaque formation and improved cognitive performance [10]. Similarly, temporary ablation of microglia with PLX5622 reduced neuroinflammation, neurodegeneration in traumatic brain injury model with notable neurobehavioral improvement [40]. Engineering hematopoietic stem cells to deliver glial cell line derived neurotrophic factor (GDNF) directly to sites of neurodegeneration in mouse models of PD to mitigate decline in motor function, comorbid cognitive impairment and depressive-like behaviors [41]. These examples indicate that targeted strategies with strong evidence from preclinical studies will pave the way for development of specific treatment for CNS disorders.

## 3. The Kynurenine Pathway

**Figure 1:**
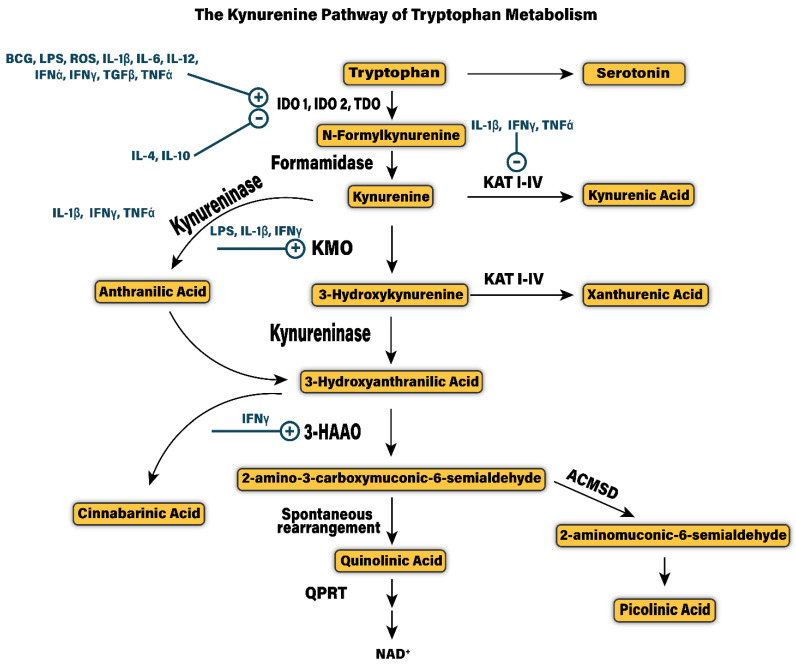
The Kynurenine Pathway of Tryptophan Metabolism. The schematic provides a comprehensive overview of the KP metabolism with metabolic breakdown products produced by oxidative and reductive actions of associated enzymes. Regulation by cytokines, damage and pathogen associated molecular patterns on KP enzymes are denoted with ‘+’ or ‘–’ in the figure [46,47,48,49,50,51,52].

The essential amino acid l-Tryptophan (trp) serves as the precursor for the synthesis of several indole related compounds like serotonin, melatonin and kynurenine that are essential for growth, metabolism and longevity [6,42]. Outside of the brain, 95% of trp is broken down to kynurenine while less than 2% of all trp is converted to the neuroactive compounds serotonin and melatonin [43]. The enzymes, IDO1, IDO2 and tryptophan 2,3-dioxygenase (TDO) catalyze the oxidative cleavage of the indole ring of trp to convert it to N-formylkynurenine [44]. Another enzyme, kynurenine formamidase or kynurenine formylase then rapidly hydrolyzes this intermediate product to kynurenine [45]. In the periphery, TDO is responsible for hepatic kynurenine production, whereas IDO is the rate-limiting enzyme for kynurenine synthesis outside the liver. Following the production of kynurenine, KP metabolism segregates along two primary branches and several smaller branches that give rise to the production of physiologically and neurochemically active kynurenine metabolites. Figure 1 illustrates the KP of tryptophan metabolism and its regulation by cytokines.

In peripheral tissues including liver and kidney, phagocytes like monocytes and macrophages catabolize the majority of kynurenine along an oxidative branch initiated by the enzyme kynurenine-3-monooxygenase (KMO) (also known as kynurenine 3-hydroxylase) that adds a hydroxyl group to kynurenine converting it to 3-hydroxykynurenine (3-HK) [53]. In the brain, microglia cells predominantly breakdown kynurenine to 3-HK as astrocytes lack the enzyme KMO required for this step [54,55]. Oxidative cleavage of 3-HK by kynureninase generates 3-hydroxyanthranillic acid (3-HANA) which is the precursor to QA, a potent neurotoxin [56]. The enzyme 3-hydroxyanthranillate-3,4-dixogygenase (3-HAO) catalyzes the conversion of 3-HANA to QA [57]. In peripheral tissues and the brain, the enzyme quinolonate phosphoribosyl transferase (QPRT) metabolizes QA to form NAD that facilitates energy production [58]. Within the mammalian brain, the enzyme capacity of QPRT is limited and becomes the rate-limiting step in NAD synthesis keeping the production in check. The reaction also generates a highly unstable intermediate product 2-amino-3-carboxymuconic-6-semialdehyde (ACMS) with a half-life of approximately 20 min and spontaneously rearranges to form QA [59]. Furthermore, the enzyme ACMS decarboxylase generates another by-product known as picolinic acid (PA). In the human CNS, both glial cells as well as neurons produce PA and may play a neuroprotective role.

The second major branch of the KP occurs by irreversible transamination of kynurenine to generate kynurenic acid (KA) that acts as a neuroprotectant under basal conditions. The enzyme, kynurenine aminotransferase (KAT) catalyzes this reaction, and four different KATs (KAT I–IV) have been discovered in mammals. These include KAT I/glutamine transaminase K/cysteine conjugate beta-lyase 1, KAT II-aminoadipate aminotransferase, KAT III-cysteine conjugate beta-lyase 2 and KAT IV-glutamic-oxaloacetic transaminase 2-mitochondrial aspartate aminotransferase [60]. In the brain, astrocytic KAT II is the predominant enzyme responsible for KA production [61].

Notably, in the brain, and alternate route of oxidative kynurenine metabolism can bypass KMO formation of 3-HK. The enzyme kynureninase can also metabolize kynurenine to generate anthranilic acid that can act as a better substrate for QA production in the brain [62]. 3-HK and 3-HANA also can serve as substrates to generate additional by-products possessing unique neuroactive properties and discussed in Section 4. Xanthurenic acid (XA) is produced by the transamination of 3-HK, catalyzed by KAT II and recently reported to possess neurotransmitter activity at metabotropic receptors in the nervous system [63,64]. Downstream of 3-HK, the dimerization of 3-HANA, i.e., condensation of two molecules of 3-HANA produces cinnabarinic acid (CA). Studies have found that CA is present in liver, kidney, spleen, lung and the brain [65,66].

Under physiological conditions, the actions of different enzymes, vitamins and co-factors, compartmentalization of metabolism and excretion through the renal system tightly governs KP metabolism [53]. From the periphery, the precursor tryptophan, kynurenine and 3-HK are the only metabolites along the KP that cross the blood brain barrier (BBB) through the large neutral amino acid transporter [66]. In addition, anthranilic acid crosses the BBB by passive diffusion to appreciable levels. The other major metabolites including 3-HANA, KA and QA poorly diffuse across the BBB. Thus, the de novo synthesis of these metabolites depends on the enzymatic activity in the glial cells and neurons [55,67]. Several clinical studies have found the increase in kynurenine/tryptophan (K/T) ratio in the periphery to be associated with CNS diseases and serves as a reliable biomarker to highlight dysregulation in KP metabolism [68,69]. Furthermore, this ratio is an important indicator of IDO activity, the critical enzyme that regulates tryptophan breakdown to kynurenine during inflammation. Importantly, IDO is stimulated in the body by growth factors, cytokines and steroid hormones [70,71]. However, under inflammatory insults increased production of pro-inflammatory cytokines like interferon, challenge with infectious agents and several diseased states, the activity of IDO is upregulated that disproportionately increases the level of kynurenine in the circulation and in brain tissue [72,73]. IDO upregulation in the periphery occurs in immune system derived cells like dendritic cells, monocytes and macrophages that respond to immune activation [73]. The majority (>60%) of kynurenine in the brain is directly transported from peripheral circulation [74]. An increase in circulating K/T ratio can cause an increased flux of kynurenine across the blood–brain barrier due to concentration-dependent competition for the large neutral amino acid transporter, and during pathological CNS conditions the BBB can become leaky to increase passive transport [14,75]. Similarly, the enzyme KMO is also upregulated by immune stimulation and disease state to increase the oxidative metabolism of kynurenine towards the production of QA in microglia and, when unchecked, contribute to increased neurotoxicity [76]. Unlike IDO and KMO, the enzyme KAT is not induced or upregulated due to inflammation, which shifts the balance between QA and KA that is critical for maintaining KP metabolism homeostasis. In addition, interferon gamma (IFN-γ) mediated IDO induction is potentiated by the action of TNF-α, IL-1α, Toll like receptors, pattern associated damage patterns or memory recognition cells of the immune system, that all increase NF-κB dependent signaling [77]. Sustained hyper-activation of NF-κB further dysregulates immune signaling due to changes in the profile of immune genes, growth factors, developmental genes, hormonal and homoeostatic signaling. Immune cells of various types exist in the CSF, meninges and parenchyma that further contribute to increased KP metabolism and its metabolites in the CNS.

## 4. KP Metabolism, Immune Cell Trafficking and Neuroimmune Signaling

The antiquated idea that the brain is an immuno-privileged organ devoid of active inflammatory processes has been replaced by new understanding that the CNS has dynamic and robust, albeit unique and very tightly regulated, immune activity. Several CNS disease models have reported enhanced trafficking of immune cells as well as dysfunctional signaling of existing immune and glial cells [14,78,79]. Immune cell trafficking to the brain serves important roles as resident immune cells, microglia and infiltrating immune (leukocytes, neutrophils, T-cells) perform critical roles like clearing debris and apoptotic cells, enhance repair in areas of injury and produce growth factors for trophic support, synaptic pruning and immune surveillance among other functions. However, inflammatory conditions and diseased states cause BBB leakiness, disruptions in tight junction, adhesion molecules and enhanced transport of cytokines and metabolites that disrupt normal brain function. In the brain perivascular spaces, endothelial cells (EC) and pericytes have the machinery for KP metabolism. While EC’s constitutively produce KA and perciytes produce PA, immune activation by inflammatory cytokines like IFN-γ and TNF-α increase the production of kynurenine through these cells [80]. Under normal and infectious condition, IDO activity in brain endothelial cells serve to limit lymphocyte proliferation and prevent brain damage by metabolizing dietary tryptophan to kynurenine that has anti-microbial and immunomodulatory functions [81,82]. In CD8^+^ T cells, IDO is an immunoregulatory enzyme role and plays an immunosuppressive role that is important in adaptive immune responses [83]. CD8^+^ T cells response are critical in mitigating the effects of viral infections like HIV or *Toxoplasma gondii* by clearing virus-infected cells [84]. Recent evidence indicates that hyper-activation of IDO in the brain could be responsible for decreased proliferation of CD8^+^ T cells, increase cytotoxicity by impairing mitochondrial bioenergetics and negatively regulate inflammatory signaling [84]. Alterations in adaptive immune signaling result in significant immunosuppression and risks the organism to opportunistic infections resulting in premature death. Zang et al. recently observed an increase in myeloid cell infiltration in the mouse brain, after treatment with kynurenine that have CD45^hi^CD11b^+^ signature in addition to astrocyte activation [85]. Further, treatment with kynurenine enhanced the chemotactic activation of peripheral monocytes, which furthers the crosstalk between peripheral immune cells and glial cells in an in vitro co-culture system via kynurenine-aryl hydrocarbon receptor (AhR) axis [85]. Cerebral ischemia in mice increased IDO in cerebral arterioles but inhibition with 1-MT, an IDO inhibitor did not change ischemia outcomes. Unrelated to the primary outcomes of ischemia, increased IDO activity could play a role in inducing co-morbid anxiety and depression observed after stroke. Indeed, clinical reports from ischemic patients show an increase K/T ratio and decreased ratio of 3-HANA to anthranilic acid in addition to IDO activation, increased oxidative stress and increased glial cell activation [86]. Interestingly, inhibition of KMO in rodent models of cerebral ischemia did reduce infarct volume and improved functional outcomes [37]. Given this observation, one would expect that IDO inhibition would also exert beneficial therapeutic effect in stroke models. However, it may be the case that TDO, rather than IDO, is driving the KP metabolic response, or our lab has reported that KMO inhibition results in kynurenine accumulation and has negative regulatory activity on microglial activation [87]. This finding introduces the possibility that not only do KP metabolites exert direct neurochemical effector activity, but they also play a previously unappreciated neuroimmunoregulatory role.

## 5. The Gut-Microbiota-Brain Axis and KP

Trp is the precursor for the synthesis of both serotonin and kynurenine. An emerging literature implicates dysregulation of gut microbiota and the related gastro-enteric nervous system in the pathology of the highly co-morbid irritable bowel syndrome and neuropsychiatric conditions depression, anxiety disorder and ASD [88,89]. In the gastrointestinal system (GI), commensal bacteria of the large intestine breakdown tryptophan and produce, several indoles and indole related compounds including kynurenines, melatonin and serotonin that are neuroactive. In the GI system, kynurenines have immunomodulatory properties, antimicrobial properties and germ-free mice show reduced Trp metabolism along the KP in addition to deficits in the innate immune system [90]. Germ free adult mice show structural alterations in amygdalar and hippocampal neurons, the areas known to be dysfunctional during stress, anxiety, depression and post-traumatic stress disorder (PTSD) [91]. Structural alterations often lead to functional changes in neurocircuitry and are important for learning and memory, long–term potentiation and long-term depression. GI inflammation activates IDO, increasing the oxidative metabolism of KP and production of KP metabolites like Kyn, KA, CA and XA that act as direct ligands to AhR [90]. Importantly, AhR signaling in the GI is critical for adaptive immunity, intestinal homeostasis and mucosal barrier function. Accordingly, mice that lack AhR show high susceptibility to infections highlighting AhR as an important mediator of cross talk between KP and the gut microbiota to regulate immune response. Upregulation of IDO during GI inflammation can alter AhR signaling by the activity of KP and dysregulate inflammatory genes like IL-6, interleukin-22 (IL-22), growth factors, prostaglandins and cytochrome P450 1A1 (CYP1A1) that are under the regulation of AhR [92]. In addition, IDO activation can also counter the balance between QA and KA, which have neurotoxic and neuroprotection properties, respectively. Dysregulated balance can affect intestinal motor or sensory function of the enteric neurons that signal via glutamate receptors with implications for the role of KP dysfunction in psychiatric conditiMCPons [93,94]. Chronic gut inflammation in mice causes depressogenic and anxiety like behaviors that are positively correlated with increased levels of TNF-α, IFN-γ, increased K/T ratio and decreased hippocampal brain derived neurotrophic factor (BDNF) mRNA [95]. Chronic stress, an important risk factor in the etiology of psychiatric disorders also alters the gut-microbiota composition with a concurrent increase in IL-6 and the monocyte chemotactic factor-1 (MCP-1) that regulate the crosstalk between peripheral and CNS immune response [96].

## 6. Brain Regional Heterogeneity in KP Metabolism

The activation of KP is associated with depressive and anxiety like behaviors in animal models [52]. Such neurobehavioral alterations orchestrate via distinct brain regions, and the effect of immune activation in the brain may be due to the role of QA and KA in modulating glutamatergic neurotransmission by acting as N-methyl-D-aspartate receptor NMDAR agonists and antagonist, respectively. Recently, Parrott et al., observed differential oxidative neurotoxic KP metabolism in nucleus accumbens, amygdala, dorsal and ventral hippocampus with dorsal hippocampus especially vulnerable to the effects of systemic LPS administration [97]. The same group also noted that an increased 3-HK/KA ratio indicative of increased metabolism in the hippocampus (dorsal + ventral) compared to other brain regions like amygdala and nucleus accumbens [98]. In a transient ischemia model, Heyes and colleagues observed increased QA in brain regions like the thalamus, cortex and hippocampus; however, the levels in hippocampus were higher when compared to the thalamus [99]. Similarly, Soo Koo et al., recently showed elevated IDO-1 expression after ischemic stroke in the nucleus accumbens, hippocampus and hypothalamus but not in the striatum with concurrent increase in 3-HAAO, QA and ROS in these brain regions [100]. Imaging tryptophan metabolism in brain tumor associated depression patients, however, show abnormal tryptophan metabolism in the fronto-striatal network encompassing the frontal cortex, striatum and thalamus that is present in both ipsilateral and contralateral hemispheres [101]. Understanding the functional relevance of brain region specific KP metabolism is still incomplete. Differences between pre-clinical and clinical models, methodological differences and brain region heterologous expression system of nerve and glial cells may account for significant variances in the observations related to KP metabolism. Further research in this area is required to delineate accurate differences in KP metabolism profiles in different brain regions. The discovery of differential KP metabolism and its activity in heterologous brain structures will greatly enhance the understanding of the contribution and extent of KP metabolism in within the different brain regions.

## 7. KP Metabolites and Molecular Mechanisms

### 7.1. Kynurenine

Oxidative cleavage of l-trp catalyzed by IDO1, IDO2 and TDO produces kynurenine, the first metabolite along the KP [44]. A consistent finding replicated across pre-clinical and clinical studies involving trp metabolism is the increase in the production of kynurenine in peripheral and brain cells alike during immune activation. The production of downstream KP metabolites depends on kynurenine and therefore the activity of enzymes IDO1, IDO2 and TDO is rate limiting in determining the potential contributions of KP in CNS inflammatory conditions. Recent discoveries that kynurenine is an endogenous ligand to AhR, a xenobiotic receptor and transcription factor and can act as a vasodilator in endothelial cells has renewed interest in the physiological role of kynurenine previously considered physiologically inert. Acting as an agonist at AhR, a transcription factor for several genes places Kyn-AhR axis to serve immunomodulatory role (Table 1) as AhR signaling is critical for immune tolerance, tumor evasion, cell adhesion and migration [102]. As such, Cuartero and colleagues recently found l-kynurenine to activate the AhR pathway in the brains of a mouse model of stroke and contribute to infarct volume in a Kyn-AhR dependent fashion [103]. In bone marrow mesenchymal stem cells, kynurenine application disrupts autophagy and induces senescence which could be blocked by the use of AhR antagonist indicating that the two phenomena are related [104]. Using AhR null mice, Lara et al., found increased levels of KAT II and KA that served to be neuroprotective in mice injected intrastriatally with the excitotoxic agent QA along with reduced lipid peroxidation and ROS formation compared to wild type mice [105]. Moyer et al., also found beneficial effects inhibiting IDO1 and AhR activity on diet-induced obesity that is related to TLR2/4-TGF-β-NF-κB signaling network [106]. In a glioblastoma cell line-U87MG, inhibition of IDO1/TDO with RY103, Kyn-AhR mediated proliferation of glioma cells reduced that suggest Kyn-AhR involvement in pathology of glioblastomas [107]. In dendritic cells with exposure to LPS or IFN-γ, Kyn-AhR signaling is involved in conferring tolerogenicity in these cells [108,109]. In fact, N-acetlyserotonin was recently discovered as a positive allosteric modulator of IDO-1 and abrogated neuroinflammation in an experimental autoimmune encephalomyelitis (AE) model by engaging IDO-AhR signaling [110]. Additionally, IDO1-Kyn-AhR axis is involved in maintenance of human embryonic stem cells in an undifferentiated state [111]. Given the important roles of AhR in immunomodulation, tumor evasion and cell cycle biology, future studies focusing on inflammation mediated Kyn-AhR signaling demand attention as a potential therapeutic target for brain tumors and neuroinflammation. Kynurenine function with respect to oxidative stress and ROS formation is unclear as studies have reported both pro-oxidant and antioxidant properties [112,113]. Such differences in the actions of kynurenine on oxidative stress maybe related to differences in in vitro and in vivo models, methods of inducing oxidative stress and the relative contributions of the enzymes in different tissue systems that produce kynurenine.

### 7.2. 3-Hydroxykynurenine (3-HK)

Downstream of kyn, the enzyme KMO produces 3-HK, a pivotal branching point along the KP. Similar to kyn, 3-HK is able to cross the BBB by engaging the large neutral amino transporter. Within the brain, cellular uptake is by sodium-dependent transporters with the highest in cortical areas followed by subcortical areas like striatum and hippocampus with the least observed in cerebellum [135,136]. In fact, 3-HK is more toxic to cells in the cortex and striatum versus cerebellar granule cells [118]. Elevated levels of 3-HK have been a common finding in CNS inflammatory disorders [118,137]. However, the exact contributions of 3-HK in neuropathology remain poorly understood mainly due to relatively low number of in vivo studies that directly evaluate its contribution and mechanisms of pathology. 3-HK is known for both pro-oxidant and antioxidant properties [112]. While oxidation of 3-HK generates superoxide anion and hydrogen peroxide that contribute to 3-HK mediated toxicity of neurons, 3-HK can also act as a free radical scavenger and can reduce lipid peroxidation [119,120,138]. This dual role could be explained by the relative concentrations of 3-HK in the cell as well as the redox state of the cell. Accordingly, Gonzalez et al., found 3-HK to have pro-oxidant like effect when used in lower concentrations whereas a higher concentration induced anti-oxidant effect that was related to stimulation of glutathione-s-transferase, superoxide dismutase and nuclear factor erythroid-derived 2-like 2 (Nrf2), a transcription factor important for antioxidant regulation [139]. In vivo experiments involving 3-HK administration in the striatum of rats showed a time and concentration dependent effect of 3-HK in increasing lipid and protein oxidation acutely that resolves within days [139]. Perhaps, 3-HK’s dual role may be important for the physiological maintenance of cellular homeostasis reviewed here in detail [118]. It is important to highlight that the effect of 3-HK in mediating neurotoxicity seems to be independent of QA-NMDA receptor interaction dependent toxicity [140,141]. However, sustained immune activation related alterations in KP metabolism and reports from clinical studies that note increased levels of 3-HK do suggest pathological roles. In mice, direct administration of 3-HK dose-dependently precipitated depressive-like behaviors and cognitive impairment. Concurrent increases in 3-HK through microglia, alterations in redox cellular sensing mechanisms during disease and inflammatory states and simultaneous deleterious effects of other KP metabolites might synergize to induce cytotoxicity [98]. In particular, the ratios of 3-HK/KA and 3-HK/QA are important biomarkers to assess neuropathological contributions of KP metabolism.

### 7.3. 3-Hydroxyanthranillic Acid (3-HANA)

The precursor to QA, 3-HANA is derived from the oxidative cleavage of 3-HK by kynureninase or by the action of non-specific oxidases on anthranilic acid that convert it to 3-HANA. Studies evaluating the role of physiological effects of 3-HANA have found both pro and anti-oxidant properties as well as anti-inflammatory properties. Decreased levels of this intermediate KP metabolite are present in blood of patients that suffered a stroke, had a chronic brain injury or coronary bypass, but levels are higher in patients suffering from Huntington’s Disease (HD) or depression [142]. The ability of 3-HK and 3-HANA to generate superoxide and hydrogen peroxide is copper-dependent, and both 3-HK and 3-HANA enhance copper associated toxicity [143,144]. Like 3-HK, 3-HANA’s effect in promoting apoptosis in monocytes stimulated by IFN-γ is related to the generation of hydrogen peroxide when 3-HANA undergoes oxidation and treatment with anti-oxidants reduces the apoptotic effect [114]. Studies employing cultures from human fetal nervous system documented anti-inflammatory and anti-oxidant properties of 3-HANA that are related to inhibiting cytokine and chemokine expression and elevating the expression of the anti-oxidant enzyme hemeoxygenase-1 [145]. Remarkably, the effects persist in both astrocytes and microglia, the glial cells most involved in regulating the inflammatory response in the CNS. In addition, 3-HANA is attributed with nitric oxide scavenging properties [115]. Recently, a study found 3-HANA to disrupt mitochondria mediated energetic metabolism not related to ROS formation under in vitro and in vivo experimental conditions [146]. Thus, similar to 3-HK, 3-HANA’s ability to play a dual role may be dependent on the redox state of the cell or model itself. Other studies pertaining to 3-HANA in cardiovascular biology and lipid metabolism suggest potential involvement of 3-HANA in cardiovascular function. Berg et al., using low density lipoprotein receptor (Ldlr) knockout mice and 3-HAAO inhibition show that increasing 3-HANA levels decreases inflammasome activation in macrophages and decrease hyperlipidemia in this mouse model of obesity suggesting 3-HAAO as a novel target for combatting cardiovascular disease, one of the biggest risk factors for stroke patients [147]. Previous research evaluating the role of 3-HANA in its ability to decrease lipid peroxidation, inhibit LDL, decrease plasma levels of lipid and decrease atherosclerosis giver further validity to the findings reported by Berg and colleagues [116]. Finally, 3-HANA is involved in modulating the proliferation of T-cells that involve AhR-NF-κB signaling in dendritic cells [117].

### 7.4. Quinolinic Acid (QA)

One of the major metabolites in the KP that has been the subject of intense research interest is QA that is produced by the enzymatic breakdown of 3-HANA by 3-HAAO. Of all KP metabolites, the evidence implicating the involvement of QA in the pathology of neurodegenerative, neurological and neuropsychiatric problems remains strongest. Early studies established QA as an endogenous glutamate receptor agonist acting on NMDA receptors [148]. QA is excitotoxic as intracerebroventrically (icv) injections of QA induce epileptic phenotype in mice and cause axon sparing lesions on neurons [56,148]. In the brain, microglia are responsible for the production of QA and represent the major source as astrocytes and neurons do not possess the machinery required to produce QA although they can uptake it for production of NAD [54,149]. Elevated levels of QA are a common finding among patients suffering from HIV infections, in neurodegenerative disease like AD, PD and HD. Injections of QA in striatum replicate the structural and neurochemical pathology observed in HD, an observation that made it a model to induce HD in laboratory settings [150,151]. In conditions like stroke and traumatic brain injury, over-activation of IDO1-mediated oxidative KP metabolism and increased QA levels contribute to secondary damage associated in the peri-infract area and have been postulated to contribute to post-stroke depression [100,152]. Administration of the KMO inhibitor Ro 61-8048 decreases the infarct volume caused by experimental ischemia [153]. In psychiatric conditions including severely depressed patients with suicidal ideation, levels of QA have positively correlated with symptom severity [154]. QA induces tau phosphorylation in human neurons, and tau associated neurofibrillary tangles are considered a pathological hallmark of several dementias and AD [155]. Moreover, the other major pathological feature, Aβ plaques activate human macrophages and microglia which enhance the production of QA. High immunoreactivity against QA is another feature observed in the AD brain [149].

At the neurocircuitry level, non-lethal QA injections induce learning and memory deficits that are dependent on the fronto-cortical and cortico-limbic areas of the brain [155,156]. Importantly, deficits most commonly observed in neurodegenerative and psychiatric disorders often arise due to malfunctioning of the neural circuits that depend on these brain regions. QA impairs spatial learning that depends on the hippocampus and chronic QA decreases functional connectivity between prefrontal cortex and the hippocampus [156]. Recently, Parrott et al., utilizing 3-HAAO knockout mice have shown that these animals are protected against LPS induced changes in behavior that depend on hippocampus suggesting neuroprotective effects of abolishing QA production in the hippocampus [97]. AD pathology usually emerges in hippocampus eventually affecting cortical areas, and lower cortical and hippocampal volumes are observed in MDD patients, which correlate with neurotoxic and neuroprotective branches of KP [157]. At the molecular level, QA is a relatively weak agonist at NMDAR that shows high binding preference for NMDARs containing NR2A and NR2B subunits [158]. The forebrain areas are highly susceptible to damage by QA as these regions have the highest amount of NMDARs with these subunits [46]. In addition, QA perturbs actin-cytoskeleton dynamics in neurons and astrocytes which disrupts normal protein transport required for maintaining synaptic homeostasis [159]. Moreover, QA is known to increase oxidative stress by generating free radicals and increase lipid peroxidation [131]. Aside from the direct effects of QA on neurons by acting as an NMDAR agonist, QA contributes in activation of glial cells and upregulates chemokine production namely MCP-1 and expression of the related chemokine receptors that is similar to action of pro-inflammatory cytokines TNF-α, IL-1β and IFN-γ [160]. Likewise, QA has other non-NMDAR mediated effects that include induction of neuronal apoptosis, lesioning and death of oligodendrocytes, production of free radicals that increases ROS formation and cause lipid peroxidation, impair mitochondrial respiration that have been reviewed in detail previously [46]. Inflammatory stimuli activate immune cells in the periphery and upregulate the oxidative branch of KP that increases QA production in macrophages and microglia. CNS associated inflammatory conditions where the BBB is leaky, infiltration of peripheral macrophages that are able to produce higher amounts of QA compared to microglia are another source of this neurotoxic metabolite adding the fuel to the fire.

### 7.5. Kynurenic Acid (KA)

One of the most important metabolites from a therapeutic standpoint is KA, produced by the irreversible transamination of kynurenine by the enzymes KAT I-IV [60]. In the brain, the synthesis of KA happens de novo in astrocytes by the enzyme KAT II after kynurenine uptake by the large neutral amino acid transporter [161]. Aside from KATs, KA may be synthesized from other sources in the body that are reviewed by Ramos-Chávez et al. [162]. KA is a non-competitive antagonist at the NMDA receptors where it can bind to the glycine co-agonist site of this cation channel receptor, an antagonist at the α_7_ nicotinic acetylcholine receptors (α_7_nAChR) and also activates the orphan G-protein coupled receptor 35 (GPR35) [123,163]. The effects of KA in the brain are varied and can modulate glutamatergic, acetylcholinergic, gamma aminobutyric acid (GABA) and dopaminergic neurotransmission [164,165,166]. As an antagonist to excitatory glutamatergic receptors, low amounts of KA increase α-amino-3-hydroxy-5-methyl-4-isoxazolepropionic acid (AMPA) receptor response, at higher concentrations, KA can act as an anti-convulsant blocking the excitotoxic effects of glutamate, kainate and QA [124,125]. Application or local administration of KA decreases glutamate output whereas blockade of KA synthesis increases glutamate release exhibiting bi-directional control over glutamate neurotransmission; an effect that is likely dependent on KA inhibiting α_7_nAChR [167,168]. Alterations in KA are associated with schizophrenia physiopathology, elevated levels of KA are found in CSF and cortical areas along with reduced KMO activity that suggests imbalance between the two main branches of KP [169]. Glutamate neuromodulation by the action of KA on α_7_nAChR is involved in the regulation of cognitive flexibility governed by medial prefrontal cortex (mPFC). Injections of kynurenine increases KA production in the brain and cause cognitive dysfunction in the attention set-shifting task governed by the mPFC circuits and administration of galantamine, a positive allosteric modulator of α_7_nAChR reverses the impairments [170]. Moreover, KA mediated blockade of presynaptic α_7_nAChR decreases the inhibitory GABAergic component in prefrontal cortex and hippocampus, which imbalances the excitatory-inhibitory balance of synaptic transmission and may contribute to the cognitive deficits in schizophrenia [166,171]. It is noteworthy to mention that the effects on KA on nicotinic receptors is controversial and remains an area of active investigation; a detailed account on the evidence, support and debate on KA-nicotinic receptor interaction can be found here [127]. Oral administration of KAT II inhibitors, BFF816 and PF-04859989 block the production of KA that attenuates inhibition of glutamate release in prefrontal cortex and improve cognitive deficits that arise due to excess KA in the brain [172,173]. Using KAT II knockout mice, Potter et al., report these mice to exhibit lower levels of KA in the brain and this reduction increases glutamate release in the extracellular space, amplifies long term potentiation in the hippocampus and improves cognition compared to control mice [174]. Elevations in endogenous KA disrupts sensorimotor gating, a deficit commonly observed in schizophrenics who have higher levels of KA that potentially contribute to this schizophrenia symptom [175,176]. Patients suffering from bipolar disorder (BD) also have elevated levels of KA in the CSF; a subset of such patients who have ongoing depressive symptoms have lower KA levels in the plasma but not in the CSF suggesting pathophysiological changes are associated to brain KA production [177,178]. Activity of KA on GPR35 located on astrocytes also decreases calcium influx in these cells; decrease in calcium transients alters synaptic glutamate release, decreases synaptic currents recorded from CA3-CA1 synapses in the hippocampus [179]. Thus, KA action on GPR35 could represent another mechanism for inhibition of excitatory transmission and regulate neuronal excitability. KA negatively contributes to learning and memory process especially those related to cortico-limbic circuits [180,181]. Activation of KP by immune stimuli elevates cortical KA and produces deficits in working and reference memory [182]. The literature regarding the levels of KA in neurodegenerative disease like AD is mixed with some studies reporting differences in KA between AD patients and controls [183,184]. Several phenomenon may be responsible for these discrepancies such as differences in epidemiological characteristics, different analytical assays, plasma v/s CSF and brain measurements and differences in experimental design (sample size, concomitant drug use, inclusion and exclusion criteria, statistical tests) used to characterize and correlate differences in the KP. However, one of the more common observation among AD studies is the altered K/T ratio that is consistently high and remains perturbed indicating alterations in KP are a prominent feature of neurodegenerative diseases [185,186].

At the cellular and molecular level, KA inhibits the cytoskeleton perturbations in striatal neurons that arise due to the excitotoxin QA [187]. In addition, KA mitigates the increase in nrf2, a key transcription factor regulating oxidative stress defense thereby mitigating the effects of ROS, lipid peroxidation, protein damage and mitochondrial respiration disruption caused by application of QA on striatal slices [187]. Besides activity on CNS receptors, KA is also an agonist at the AhR, which as discussed earlier is important in immunomodulation. KA is also an anti-oxidant; reduces oxidative stress due to ROS formation, decreases protein and lipid damage.

### 7.6. Xanthurenic Acid (XA)

Xanthurenic acid is formed by the transamination of 3-HK by the KAT enzymes. As XA cannot cross the BBB, the bioneosynthesis occurs in the brain. KAT II produces XA in astrocytes and has been found in the presynaptic terminals of neurons [188]. Additionally, organic anion transporters are able to uptake XA, a finding reported from a study using oocytes of *Xenopus laevis* expressing human and rat transporters [189]. XA can act as an agonist at metabotropic glutamate receptors mGLUR2 and mGLUR3 [63,190]. Neal and others have suggested vesicle glutamate uptake transporter (VGLUT) inhibition as the mechanism instead that reduces the glutamate uptake at the presynaptic junction which leads to decreased glutamate release during synaptic stimulation [132]. Future studies are required in order to substantiate and validate either of the claims. In the hippocampus, XA decreases synaptic excitability, depresses synaptic transmission, and acts as an anti-convulsant, which could be a potential therapeutic target to resolve the symptoms of patients suffering from schizophrenia, episodes of psychosis, or epilepsy [63,64,133]. Preclinical studies support this premise as modulators of mGLUR may be beneficial in mitigating cellular and behavioral correlates of psychosis and schizophrenia [191,192]. Recently, Kubicova and researchers show that XA may act as an anti-oxidant [134]. Fazio et al., have also shown XA to act as an endothelium derived relaxing factor with the ability to induce nitric oxide synthase that could be important in the etiology of inflammation-induced hypotension. Importantly, KMO inhibitors Ro-618048 and UPF 648 were able to mitigate this endotoxin-mediated hypotension [193].

### 7.7. Cinnabarinic Acid

Oxidation of 3-HANA leads to the production of CA. This reaction creates free radical superoxide anion and hydrogen peroxide as outlined earlier and contributes in liver injury by inhibiting mitochondrial respiratory chain [194]. Accordingly, CA induces apoptosis of mouse thymocytes due to ROS formation and activation of caspase that promote apoptotic cell death. Fazio et al., reported CA to be a weak orthosteric agonist at mGLUR4 but without activity at other subtypes belonging to this family of metabotropic receptors [122]. Pharmacological application of CA at low micromolar concentrations can be neuroprotective as it reduces neuronal toxicity induced by 1-Methyl-4-phenyl-1,2,3,6-tetrahydropyridine (MPTP); however, the physiological levels found in the brain are low but elevate significantly after immune stimulation [122]. CA can also act as a ligand to AhR, thereby contributing to immunomodulation by promoting T-cell differentiation, and play a role in reducing neuroinflammation [65]. In an experimental model of AE and using mGLUR4 knockout mice, CA was able to boost the immune response, increase T regulatory cells, and reduce neuroinflammation. This could be of potential therapeutic value for the treatment of M.S. [65]. CA-induced AhR signaling is also critical for histone H4 acetylation and may serve to protect hepatic cells due to chemical insults [195].

### 7.8. Picolinic Acid (PA)

The enzyme ACMS decarboxylase (ACMSD) converts the unstable intermediate product of breakdown of 3-HANA to PA as a side chain reaction over the non-enzymatic conversion of 3-HANA to QA. The levels of ACMSD in the brain are low and when ACMSD is saturated, the non-enzymatic conversion of 3-HANA to QA predominates. Moreover, the concentration of PA is higher in the periphery due to higher ACMSD activity in the liver and kidney, and PA has low BBB permeability due to its hydrophilicity [59]. Brain EC are able to produce PA when stimulated by cytokines [80]. The levels of PA in the developing brain are low, peak in adulthood, and tend to go down with aging [196]. The physiological roles of PA are reviewed here [197]. Accordingly, PA has been shown to have anti-viral and anti-microbial properties as it can induce cell cycle arrest at the G1 stage of replication in cultured cells [128,129]. Besides, PA is an efficient metal chelator of Zn^2+^ and Fe^2+^ ions and this ability may contribute to its anti-microbial like properties [197]. PA also induces the activation of macrophages by enhancing IFN-γ dependent nitric oxide synthase (NOS) expression that accompanies expression of macrophage inflammatory proteins MIP1α and MIP1β [198]. PA disrupts T-cell differentiation and may play an immunosuppressive role by inhibiting cell cycle and metabolic activity [199]. When injected icv but not subcutaneously, PA decreased the threshold for seizures in mice although the precise mechanism of this effect is unknown [125,130]. Similarly, other studies have noted high dose injections of PA to cause toxicity in hippocampus, substantia nigra and striatum but when co-injected with excitotoxicants like QA or kainate, PA decreases toxicity [125,200]. Taken together, these findings suggest that PA could have modulatory actions on glutamatergic neurotransmission, which depends on the concentration of PA as well as the presence of other glutamate agonists like kainates [201]. It is attractive to speculate that increased amounts of local PA in the brain could saturate ACMSD due to increased substrate availability, which would shift the metabolism of 3-HANA towards production of QA, a known epileptic agent [202]. Brundin and colleagues have found a single nucleotide polymorphism in the gene ACMSD in suicide attempters that is associated with decreased ACMSD activity and corresponding low levels of PA in circulation, in addition to a lower PA/QA ratio [154]. Recently, a group of researchers found elevated levels of PA after electroconvulsive treatment in severely depressed patients who had lower serum levels of PA before therapy suggesting PA could be neuroprotective [203]. In summary, PA may confer anti-inflammatory and neuroprotective actions that could be important for neuroinflammatory, neurodegenerative and severe psychiatric conditions.

## 8. Translational and Therapeutic Considerations

**Figure 2:**
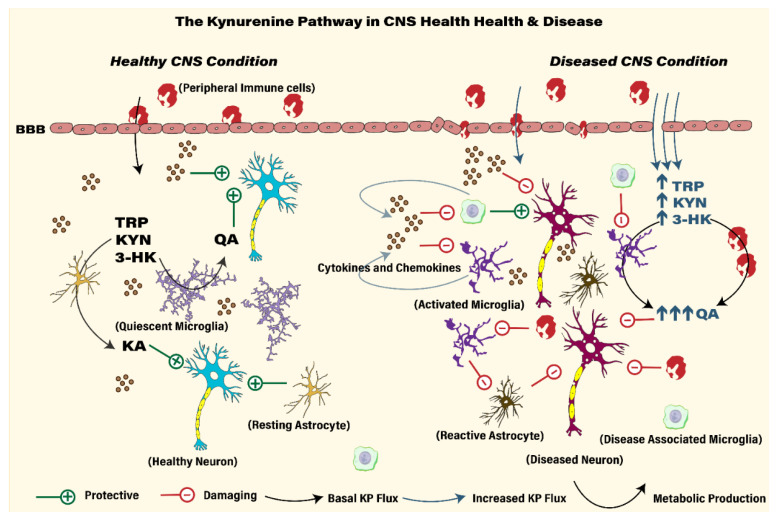
KP Metabolic Balance in CNS Health and Disease. Under healthy conditions, the intact BBB limits the entry of metabolites, xenotic material and non-CNS cells tightly regulating brain homeostasis where KP metabolites modulate oxidative stress, neuroinflammation and neurotransmission. Under CNS disease conditions, neuroinflammatory stimuli, including systemic and local insults, disrupt physical, genetic and metabolic checkpoints. The cascade of chronic inflammation ensues allowing an enhanced flux of toxic KP metabolites in the CNS system in addition to gliosis mediated release of cytokine, chemokines and KP metabolites that fuels the fire to this vicious loop [73,78,204,205,206,207].

Under physiological conditions, amino acid related metabolism is critical for many functions throughout the body and influence various organ systems. The KP, likewise, through the generation of various biologically active metabolites is important in regulation of many essential functions including energetic metabolism, oxidative stress defense, immune system regulation both innate and adaptive, tumor evasion, xenobiotic and hormonal signaling, and synaptic neurotransmission regulation among others. Under inflammatory conditions, a vicious loop of cellular and molecular changes cascade out of control that become pathological reasons for disease if not kept under check. Critical evaluation and understanding of mechanistic contributions of KP metabolites in the pathophysiology of disease has many important translational benefits. Below, we discuss recent evidence how dysregulated KP metabolism ‘fuels the fire’ to CNS diseases and some therapeutic considerations along this pathway. Figure 2 compares and contrasts the contribution of KP in CNS health and disease.

A characteristic feature of neurodegenerative disease is the burden of inflammation that contributes to the pathology of CNS disease. Microglia and astrocytes are the primary effectors of neuroinflammation, and activation of these cells is observed in several CNS disease clusters [16,208]. Neuroinflammation is a primary reason for the activation of microglia and astrocytes that occurs through the induction and upregulation of immune genes, pro-inflammatory cytokine and chemokine production, pattern and damage associated molecular patterns, oxidative stress, increased apoptosis and dysregulated KP metabolism. Microglia, the resident immune cells of the brain of the myeloid origin perform several important functions including immune repair, surveillance and modulation, phagocytosis, metabolite clearance, CNS development, synaptic pruning, regulation of synaptic transmission, synaptic plasticity, trophic support, neuronal survival and death [209]. Microglia possess the surface signatures that are important for chemokine and cytokine signaling, pattern associatedTLRs, immune signaling, adhesion proteins, and calcium binding proteins. Microglia are highly dynamic, under resting conditions they are quiescent cells with a small non-spheroidal soma and highly ramified/branched processes that survey the neighboring areas for cellular debris and pathogens. Microgliosis arises as microglia progressively turn their highly ramified structures with an enlarged amoeboid soma that is primed for release of cytokines and other inflammatory factors [210]. Recent studies using transcriptomic analysis of microglia from diseased brains identified a distinctive phenotype known as disease associated microglia that are thought to be protective. Microglia’s functional phenotype are highly dependent on local environmental cues that inform surface receptor expression. For example, triggering receptor expressed on myeloid cells 2 (TREM2) and its downstream signaling with DNAX-activating protein of 12kDa (DAP2) signaling regulate microglia function, promotes cell survival and important for phagocytosis [211]. High risk variants of TREM2 (loss of function) identified in neurodegenerative disorders and upregulation of TREM2 gene expression in AD and other dementias suggest a beneficial role [212]. However, evidence from other studies also suggest that TREM2 function in activating microglia may be age and amyloid burden dependent. Recently, researchers confirmed a suspected hypothesis that early proliferation of microglia induces senescence programs and determines specification to disease associated microglia and contribute to AD pathology [213]. A nuanced understanding of the spatial-temporal origins and activity of DAM from additional studies will be key to facilitate further discussion.

Similar to microglia, astrocytes perform several essential functions in the CNS including trophic support to neurons, synaptogenesis, regulation of synaptic plasticity and synaptic homoeostasis, neurogenesis, metabolic regulation, maintenance of BBB and support microglia in immune monitoring [214]. Astrocytes also express cytokine and chemokine receptors and under the presence of inflammatory stimuli like cytokines, chemokines, growth factors, reactive oxygen species, and inducible NOS, they undergo cellular and structural changes that lead to astrocyte gliosis [215]. The activation of astrocytes may involve NF-κB pathway and related complement signaling to induce neuronal damage. The overexpression of glial fibrillary acid protein (GFAP) and S100 calcium binding protein B (S100B) are important correlates to assess and confirm astrogliosis and these markers have been observed in CNS disorders [214,216]. Injections of the neurotoxic AD-related Aβ-1-42 peptide in mice increases IDO activation along with cognitive deficits, depressive and anxiety like behavior. In addition, Aβ 1-42 also activate microglia (via TLR2) and astrocytes that surround them, which can further aggravate and exacerbate the neuroinflammatory response and the associated KP metabolism [217,218]. Pretreatment with 1-MT prevented the development of neurochemical and behavioral deficits in the hippocampus and cortex due to Aβ-1-42 [219]. The YAC128 mouse model of HD is known for higher IDO expression and sensitivity to NMDA mediated neurotoxicity and when IDO null mice are challenged with the neurotoxic QA, lesions in the striatum are smaller that suggest neuroprotective effects due to inhibiting neurotoxic metabolite production [220]. Inflammatory stimuli mediated IDO1 hyper-activation also reduces the survival of serotonergic neurons along with marked microglial activation, another evidence of inflammatory mechanisms contributing to pathology of depression and neurodegeneration [221]. This is in agreement with other studies that demonstrate a reduction in neuroinflammation and neurodegeneration by inhibiting KP enzymes IDO/TDO and KMO listed in Table 2. As noted earlier, QA can disrupt the cytoskeleton dynamics in neurons, cause oxidative damage and can be fatal to neurons. Evidence from the literature suggests that inhibiting microglia activation may augment neuronal survival as treatment of BV-2 microglial cells with the IDO inhibitor 1-MT, the KMO inhibitor Ro-61-8048, dexamethasone, or MK-801 prevented atrophy of cultured cortical neurons [222]. Moreover, the conditioned medium generated from QA application on BV-2 cells causes cortical neuron nuclear fragmentation and disrupts neurite growth that suggest microglia activation is critical in mediating the effects of neuroinflammation and KP metabolism [223]. As outlined earlier, QA and KA have opposing effects on glutamate neurotransmission and imbalance in the levels of QA/KA disrupts the physiological modulatory roles of these neuroactive metabolites. Taken together, reactive microglia and astrocytes act as ‘seeds’ of damage which compound the effects of neuroinflammation by initiating several catastrophic cellular and molecular phenomena that are beyond the scope of this review.

The two major contributors to KP metabolism within the brain are microglia and astrocytes that carry out either the oxidative metabolic branch or the irreversible transamination branch of KP, respectively [54,251]. Dysregulation of KP metabolism is associated with the induction and upregulation of IDO1 and IDO2 mediated oxidative breakdown of tryptophan to kynurenine. The induction and upregulation of IDO following immune system activation occurs through IFN-Ɣ dependent and independent mechanisms (IL-1β, IL-6, TNF-α, TGF-β) [49]. Upregulation of IDO-1 is a well-documented observation in CNS diseases and genetic or pharmacological inhibition studies of IDO are beneficial in modifying or reducing pathological traits associated with CNS pathology [107,252]. In AD, IDO activation is associated with senile plaques and neurofibrillary tangles in the hippocampus and cortical areas, which prime microglia and increase production of inflammatory cytokines, ROS and neurotoxic QA. During disease progression, sustained activation of these phenomena may contribute to neuronal death due to actions of cytokines, ROS, NO and QA induced glutamate excitoxicity. Animal models of AD show increased IDO1, TDO expression, higher levels of oxidative metabolites and enzymes along the 3-HK branch [149,253]. Inhibition of IDO/TDO decreases neurodegeneration, decrease accumulation of toxic KP metabolites and improve behavioral performance in learning and memory tasks often compromised in dementias [254]. IDO inhibitors are useful in improving outcomes in preclinical models of neurodegenerative, neurological and psychiatric disease. Inhibition of IDO prevents the metabolism of kynurenine down the KMO branch, thus preventing the generation and accumulation of free radical generators that induce neuronal loss. Moreover, IDO inhibition mitigates the behavioral dysfunction associated with inflammation and seizures that arise due to perturbed glutamate neurotransmission [225,227]. N-acetylserotonnin, a positive allosteric modulator of the IDO enzyme may be of value in reducing neuroinflammation associated with these disorders and recognized for its neurotrophic and anti-depressant effects by activating the BDNF—tropomyosin receptor kinase B (TrkB) signaling pathway critical in synaptic plasticity [110].

KA, as a non-competitive antagonist at NMDA receptor in the context of neurodegenerative and neurological conditions can counteract the excitotoxic effect of excess glutamatergic signaling through NMDA and non-NMDA dependent mechanisms. The class of compounds that include KMO inhibitors block oxidative metabolism towards QA production and are effective in reducing dyskinesia, motor function impairment in Parkinson models and prevented ischemia mediated neuronal damage and apoptosis [228,255]. In addition, other KMO inhibiting compounds reduce neurodegeneration, associated synapse loss and neurobehavioral dysfunction in animal models of HD and AD [230,236]. This suggests that reducing oxidative stress and preventing excessive glutamate signaling presumably due to increased KA/QA ameliorates underlying dysfunction in Parkinson’s and ischemia. Future studies should critically review using KA/QA ratio for systematic assessments of neuroprotection and vice versa for neurotoxic effects. Since KA can reduce glutamatergic neurotransmission via inhibiting NMDA and nicotinic acetylcholine receptors, KA analogues could have therapeutic vitality in preventing the effects of excess glutamate in neurological and neuropsychiatric disorders [249]. KYNA analogues listed in Table 2 may be important tools for the development of therapeutics as they have found utility in preclinical models of HD, ischemia and epilepsy by preventing aberrant epileptiform activity, prevent excessive neuronal atrophy, improve motor behavior and may aide neuronal survival [234,256].

Cytokine-associated changes in behavior associated with dysregulation KP metabolism were made in patients undergoing immune therapy for Hepatitis C infection and cancers [257]. Following immune therapy, patients reported symptoms of depressed mood, anxiety and cognitive malfunction like symptoms that could be resolved with the anti-depressant paroxetine [23]. It was not long after this that researchers hypothesized that cytokine induced disruption in KP may be responsible for the alterations in psychoneuroendocrine affective behaviors [257]. Several of these affective behaviors are associated with most currently known neurodegenerative disorders, neurological and psychiatric disorders that involve the cortico-thalamic, cortico-limbic structures and basal ganglia of the forebrain. The afferents of the vagus nerve projects signals towards the brain stem nuclei (nucleus tractus solitarius) which relays these signals to locus ceruleus, the rostral ventrolateral medulla, amygdala and thalamus that regulate emotional and cognitive behaviors in response to these signals [258]. The vagus nerve has receptors for cytokines and PAMPS released by a variety of cells in the gastrointestinal tract that communicate peripheral immune signals to the brainstem and spinal cord and transmits these signals to the hypothalamus activating the sympathetic nervous system [204]. The hypothalamus–pituitary–adrenal (HPA) axis plays critical role in the etiology of affective behaviors as they respond to peripheral inflammatory signals and stressor induced elevated glucocorticoids levels. Chronic stress and chronic activation of the immune system sustains the production of these danger signals that lead to excessive glucocorticoid signaling which should exert a negative effect on immune signaling but soon becomes resistant [259]. Chronic activation of HPA axis and elevated immune signaling over activates and upregulates KP increasing the flux of metabolism of tryptophan towards production of KP metabolites. These metabolites increase oxidative stress and ROS formation, activate immune signaling, induce protein and lipid damage, neuronal toxicity impairing several cellular functions and could be fatal especially during degenerative, autoimmune and aging conditions.

In the laboratory, challenging mice with immune stimuli through the periphery or directly injected in the brain produce similar changes in the motivational/behavioral state of animals. They exhibit anhedonia and anxiety like behavior, altered cognition, avoid physical and social interaction, reduce food and water intake exhibiting the full array of symptoms [204]. Pre-treatment with minocycline, a tetracycline antibiotic, pharmacological (1-MT) or genetic inhibition of IDO (IDO^−/−^ mice) attenuates acute and chronic inflammation-mediated changes in behavior. This reverses the increase in inflammatory mediators and normalizes K/T ratio to physiological levels [51,260]. Interestingly, treatment with anti-depressants like SSRI’s/SNRI’s and ketamine improve symptoms of depression in humans and endotoxin-mediated sickness behavior in animal models, which are positively correlated with reduction of inflammation, normalization of KP metabolism along with elevated levels of serotonin [261,262]. In addition, chronic stress that is a critical risk factor in the etiology of mood disorders precipitate similar behavioral dysfunction in animal models. Treatment with either IDO inhibitors (1-MT), anti-inflammatory drugs (minocycline, infliximab) and anti-depressants alone or in combination synergistically reverse chronic stress induced behavioral abnormalities via anti-depressants and anti-inflammatory actions in the brain [25,263]. Treatment with anti-depressants where it is effective in improving symptoms correlates well with treatment outcomes and increase KAT gene expression which increases KA production and may offer neuroprotection [248]. Animal models of chronic stress activate peripheral innate immune response and contribute in activation of microglia that are the primary source of neurotoxic KP metabolites like 3-HK and QA. Chronic stress alters glutamate neurotransmission in the frontal cortex of rats positively related to increased IDO expression and increased QA/KA ratio representing higher risk of toxicity which is reversed by treatment with anti-depressants [264]. In humans, the stress response has an inverted U shape relationship with the benefits to the body. Repeated chronic stress in which homogeneous or heterogeneous forms of stimuli persist without representing imminent danger can engage physiological systems in the body in order to adapt and defend them. However, when the stressful stimuli are not resolved, the acute alterations in neural circuit function turn chronic leading to alterations in mood and motivation.

The levels of neurotoxic KP metabolites like 3-HK, QA/KA are elevated in patients with depression and anxiety disorders. The majority of neurobehavioral symptoms in depression and anxiety arise in cortico-limbic circuits in the brain, the imbalance in levels of KP metabolites in corresponding brain regions correlate with circuit function and disease outcome. For example, higher microglial QA immunoreactivity in subgenual and anterior cingulate cortex critical in empathy, impulsivity, emotion and decision-making correlates with symptoms of depression suggesting QA release from microglia is an important pathological contributor [265]. Young et al., found in humans with MDD, hippocampus dependent autobiographical memory recall inversely correlates with KA/ 3-HK whereas recall of negative memories positively correlates with KA/QA [266]. Furthermore, KA/QA, a potential neuroprotective index, is lower in MDD patients and negatively correlates with symptoms, but a positive correlation exists with lower hippocampal and amygdala volumes [266]. Studies employing the current pharmacological treatment options for improving depression and anxiety symptoms are known to reduce the levels of 3-HK and QA while normalizing the KA/QA ratio [246]. In patients that suffer with treatment resistant depression for whom current therapeutic options can no longer provide benefits either due to poor efficacy or due to adverse side effect profile, rapid acting anti-depressants with a low abuse profile are required. In particular, treatment with NMDA receptor antagonists like ketamine improves the outcome in treatment resistant depression that have a high rate of remittance due to lack of treatment options [34]. In 2019, esketamine nasal spray received approval by the FDA for treatment resistant depression and could be of value for depressed patients with high risk of committing suicide [267]. It is becoming increasingly evident that patients suffering with depression may be clustered under two major categories, one that respond to current treatment options and have lower inflammatory profile associated with disease while the other group is associated with exaggerated inflammatory profile and treatment resistant. Recently, Haroon et al., performed path analysis and observed segregation of depression patients using correlation between treatment response and TNF-K/T levels. Their results suggests that plasma and CSF profiling of KP metabolites in depression can serve as proxy for accurate prognosis, evaluation of treatment options and highlights the potential utility of therapeutically targeting KP metabolism in depressive patients that are poor responders to existing pharmacotherapy [268]. Supplementation of anti-inflammatory therapy to reduce pro-inflammatory expression with NSAIDS, selective COX inhibitors, TNF-α antagonists, IL-6 neutralizing antibodies and growth factors like granulocyte-macrophage colony-stimulating factor (GM-CSF) with current anti-depressants on the market may provide synergistic benefits for treatment resistant inflammation associated depression [73,247,269,270,271]. Future clinical studies employing combination therapy of aforementioned options would further our understanding of the treatment response and may facilitate novel combinatorial treatment options. Apart from pharmacological interventions, other forms of therapy like electroconvulsive therapy also tend to decrease neurotoxic QA/KA, increased KYNA/3-HK and increased KA levels; indices which correlate with improvement in depression symptoms [240,272].

## 9. Single Nucleotide Polymorphisms (SNP’s) in The Kynurenine Pathway of Tryptophan Metabolism

A growing number of clinical studies have started utilizing large-scale genomic studies from patient samples to observe genetic differences that can explain certain pathological traits. As rapid advancements continue in the area of genetics and commercial biotech manufacturing, the cost of performing such massive studies is decreasing and may aid the identification and knowledge of additional disease associated SNPs more common to researchers and clinicians. SNPs occur when the correct DNA nucleotide get substituted with an incorrect one and depending where within the gene these SNPs are located, the resulting amino acid sequence may be altered. Although most SNPs do not have any effect on function, genome wide association studies have helped in identifying functional SNPs that may enhance certain individual’s response to drugs or elevated susceptibility to certain environmental factors and risk for developing diseases. Disease associated SNP’s may alter the protein coding of certain areas in the DNA that may lead to an altered protein product which may alter the physiological role of the protein. A survey of the literature to identify functional SNPs within the KP summary is in Table 3. Multiple clinical studies have reported the association of two SNPs in schizophrenia patients, rs1053230 and rs2275163, located on the KMO gene. The findings from these studies suggest that the rs2275163, C > T allele is associated with decrease cognitive scores and deficits in visuospatial memory and increased risk of schizophrenia [273,274]. Whereas the rs1053230 variant of the KMO may not influence cognitive aspects but is strongly associated with higher risk of developing schizophrenia associated behaviors [274]. Although the exact functional relevance of this KMO allele SNP is not clear, these studies indicate that carriers of these alleles may have reduced KMO activity, which could drive the flux of kynurenine metabolism towards excess KA production. Of note, one study that involved Japanese schizophrenia patients tested associations with eight identified SNPs, but none were significant [275]. Some of these observed differences between studies might be due to sample size, racial differences, statistical tests used to measure group differences and differences in testing technologies. Additionally, two studies found trends for association between the rs1053230 GG genotype of the KMO allele in patients suffering from HIV associated depression while another study failed to find any association [276,277,278]. Other studies found IDO SNPs rs2929115 and rs2929116 that may predict the outcome of treatment response. The IDO1 SNP rs9657182 is associated with cytokine induced depression whereas KAT III rs1272958 may affect KAT enzyme function in depression patients [278,279]. In Asian women suffering from postpartum depression (PPD), the polymorphisms of KMO SNP rs1053230 AG genotype had higher 3-HK/kyn ratio indicative of increased KMO activity and associated with PPD [280]. A similar study found IDO1 rs10108662 in Asian women with increased IDO activity and associated PPD development risk that carried this allele while another study that evaluated eight SNPs in KAT I–III genes did not find any associated between PPD and KAT SNPs [281,282]. Taken together, these findings make it imperative to speculate that in case of depression, the associated SNPs may drive alter the flux towards the oxidative IDO-KMO regulated KP metabolism. Three studies, a meta-analysis of GWAS for PD, a single PD patient and a family with cortical myoclonic tremor and epilepsy report SNP’s in the ACMSD gene with increased risk of motor deficits and association with higher risk of PD [283,284,285]. Brundin et al., discovered minor C allele of the ACMSD SNP rs2121337 to be associated more frequently with suicide attempters with decreased levels of PA in the CSF that is indicative of lower ACMSD enzyme activity [154]. Lower ACMSD activity would shunt the pathway towards QA production, and since QPRT enzyme saturates at nanomolar concentrations of its substrate, the increased QA could be neurotoxic [57]. This is important for neurodegenerative diseases and patients with severe depression and suicidal ideation that have higher levels of QA drugs that increase ACMSD expression or activity may be of therapeutic use.

## 10. Concluding Remarks

Therapeutic agents used in preclinical model systems often have different routes of administration compared to pharmaceuticals approved for human use. In particular, direct application of drugs into the ventricular areas can result in significant differences between findings from the clinic and the laboratory. One of the major challenges in drug design for CNS diseases is overcoming the limitations in CNS penetrance caused by the BBB. Of KP metabolites, only Trp, Kyn and 3-HK are able to cross the BBB to act as precursors for de novo synthesis of downstream metabolites within the brain. To curtail the neurotoxic effects of KP metabolism, as is required in AD, HD, MS, epilepsy and depression, therapeutic inhibition of oxidative KP enzymes may provide benefit, while development of drugs that act at important metabolic junctions could be developed to alter metabolite ratios. In order to inhibit these enzymes within the brain, the drug must be able to cross the BBB. Pro-drug approaches may provide some benefit to this cause, as in the case of AV-101 4-Chlorokynurenine, a selective glycine site NMDAR antagonist converted to 7-chloro-kynurenine that cross the BBB and can block the glycine site of NMDAR. Another recent study by Zhang et al., documented the discovery of a novel KMO inhibitor, which is a pro-drug and reduces neurodegeneration in a drosophila HD model [290]. Another strategy that is currently under investigation is dietary manipulation of large neutral amino acids, as supplementation with leucine attenuated LPS-induced depressive like behavior in mice by competitively reducing kynurenine transport into the brain [291]. Additionally, anti-inflammatory diet consisting of a variety of foods may normalize and restore the balance in KP metabolism [292].

The gut microbiota are critical in regulating the innate immune response, growth and development, metabolism and production of biologically active molecules that exert significant physiological effects throughout the body. Importantly, the brain–gut–microbiota axis represents the complex interconnected crosstalk of the microbiome, gastrointestinal and central nervous system. The bacteria in the gut produce and release GABA, the inhibitory neurotransmitter that is involved in the pathophysiology of mood disorders and increase tryptophan availability for serotonin production [88]. In a cohort of 110 patients suffering from MDD, treatment with probiotics was associated with decreased serum K/T ratio and improvement in depression scores [293]. Moreover, Moloney et al., recently discovered the role of gut microbiota microrna mir-294-5p that target KP metabolism genes and regulate it in the hippocampus; a critical area in learning and memory as well as dysregulated in multiple CNS diseases [294]. This indicates that the gut–microbiota–brain axis is an important therapeutic avenue that may hold promise for treatment of anxiety disorders and depression [293]. Besides, the gut IDO-AhR signaling axis is critical in immunomodulation and immunotolerance that may be a critical target in reducing the burden of chronic inflammation [92]. Gut bacteria sequester Trp and produce indole related compounds that are also AhR agonists [295]. Drug development strategies to sequester excess peripheral kynurenine by gut microbiota could interrupt the flux of KP precursors towards the brain. Strategies such as allosteric modulation of KP enzymes like IDO, KMO and 3-HAAO might represent exciting drug development opportunities for neurodegenerative and neuroinflammatory diseases. In particular, the recent discovery of N-Acetylserotonin as a PAM of IDO in dendritic cells that reinstated IDO activity to physiological levels in immune cells from patients with relapse remitting MS will open new doors of information regarding the therapeutic value of this compound in reducing the burden of neuroinflammation [110]. The utilization of advances in stem cell techniques and transplant in future studies to bioengineer KP enzymes in vitro aimed at normalizing KP metabolism in CNS disease patients might be another novel form of therapeutics. For neurological conditions like seizures and psychiatric disorders that have acute episodes of mania, anxieties, psychosis and suicidal ideations, development of biologicals delivered directly in the blood stream to curtail excessive glutamate signaling in the brain may be of therapeutic value.

Pharmacological and genetic inhibition studies have contributed significantly towards our understanding of KP metabolism dysfunction in CNS disease. It is important to take under consideration that several of pre-clinical studies that involve manipulation of kynurenine pathway using rodent models that may be translationally less relevant. Over the years, several whole-body KP enzyme knockout mice have let to the discovery of seminal findings that assert KP metabolism importance within the context of physiology and disease. There is a need for studies that employ tissue or region-specific knockout mice that would make it possible to study the effects of KP metabolites within a particular organ system and brain regional heterogeneity in metabolism. As the KP metabolism is diverse and produces several metabolites, compensatory increases in the genetic and protein expression of KP enzymes and metabolites might underscore some of the laboratory findings reported from these mice. As discussed throughout the extent of this review, several KP metabolites are neuroactive, compensatory changes in metabolite production since birth may alter neurotransmission, and other physiological parameters that could confound several dependent variables measured in scientific studies. The advent of powerful and sophisticated gene editing technologies including the Cre-LoxP and CRISPR-Cas9, cell and tissue specific knockdown of KP enzyme genes combined with state of the art cellular and molecular imaging techniques will allow researchers to study brain region specific heterogeneity of KP metabolism.

Preclinical studies that study neurobehavioral dysfunction arising due to alterations in KP confirm that forebrain circuits are sensitive to the effects of these metabolites. However, additional areas that provide a deeper and mechanistic understanding of cellular and molecular actions of KP metabolites in physiology and disease that are currently underway may provide better insights. One important area of study in the future should be testing the activity of KP metabolites that act as glutamate receptor agonists on synaptic and extra-synaptic NMDA receptors. Outlining this difference is critical with respect to CNS disease as synaptic and extra-synaptic NMDA receptors can contribute to both neuron survival and susceptibility to cell death [296]. Another example is the role of KP metabolites in regulation of immune response through microglia that is still developing. In particular, the role of KP metabolites in negatively regulating the inflammatory response through microglia by dampening the production of inflammatory cytokine is recent and remains to be characterized in detail [87]. Moreover, our understanding of the cellular and neuromolecular interactions of KP metabolites with nerve and glial cells remains incomplete. KP metabolite signaling studies in the areas of cancer biology and immunology have significantly advanced the contribution of the KP in tumor pathology and immune response.

Clinical findings measuring immune markers, functional brain imaging studies, and human experimental studies show robust association between KP metabolism and the pathophysiology of several brain disorders. The nature and importance of KP in maintaining various physiological functions in the body is broad. The production of NAD, an essential cofactor required for mitochondrial energetic respiration, makes KP metabolism relevant to all cells in the body. Besides, several KP metabolites have neuroactive properties and are involved in regulation and modulation of major neurotransmitter systems like glutamatergic, GABAergic, nicotinic, serotonergic and dopaminergic. Thus, while it is attractive to target this pathway from a translational perspective, the widespread diverse actions of KP metabolites in basic biology makes that prospect challenging. In addition, targeting KP will affect biology of all indole related compounds, which represents an additional drug development challenge to avoid adverse side-effect profiles of lead compounds. Potential side-effects of targeting KP are likely to affect neurotransmitter functions, gut and immune function and mitochondrial energy production [297]. Clinical trials involving the use of IDO inhibitors for gliomas that failed have been well tolerated in studies completed so far and information from currently underway are not available yet [298,299]. From a clinical biomarker development perspective, several different indices to represent the state of KP metabolism have been used and proposed. Of these, K/T, 3-HK/kyn, KA/QA and QA/KA seem to be attractive clinical biomarkers in diseases like AD, HD, CNS infections, neurovascular and neurotrauma, mood and psychiatric disorders. Changes in the levels of these metabolites and the associated enzymes are critical to study as small changes can have substantial and biologically significant effects on metabolism, neurotransmission and immunomodulation. The development of rapidly available and brain penetrant drugs continues to remain the cornerstone need of current pharmacotherapy for CNS disease, large scale omics studies and additional research on the basic biology of KP metabolites will further the quest in the discovery of targets for CNS drug development.

## Figures and Tables

**Table 1:** Summary of individual KP metabolites with known receptor targets, influence on neurotransmitter systems and other key biological functions within the context of CNS health and disease (* debated).

Metabolite	Metabolite-Receptor Target	Neurotransmitter Activity	Biological Functions	Ref
3-Hydroxyanthranilic Acid	Unknown	Unknown	Anti-Inflammatory, Oxidative stress regulation	[114,115,116,117]
3-Hydroxykynurenine	Unknown	Unknown	Oxidative Stress Regulation	[118,119,120]
Cinnabarinic Acid	mGLUR4, AhR	Glutamate	Immunomodulation	[65,121,122]
Kynurenine	AhR	Unknown	Transcription factor, Immunomodulation, Anti-Cancer, Oxidative stress regulation	[103,111,112,113]
Kynurenic Acid	α_7_nAChR, AhR, NMDAR, GPR35	Glutamate, GABA and Nicotinic	Anti-Oxidant, Immunomodulation, Anti-convulsant	[123,124,125,126,127]
Picolinic Acid	Unknown	Glutamate	Anti-convulsant, Anti-viral, Anti-microbial, Immunomodulation	[125,128,129,130]
Quinolinic Acid	NMDAR	Glutamate	Pro-convulsant, Pro-oxidant	[46,56,131]
Xanthurenic Acid	mGLUR 2/3 *	Glutamate	Anti-convulsant, Anti-oxidant	[63,132,133,134]

**Table 2:** List of pharmacological treatments targeting KP Metabolism and neuroinflammation and evaluating treatment outcomes in CNS disease models. A wide variety of therapeutic compounds have been evaluated that directly target KP enzymes or other receptor systems which limit neurotoxic KP metabolite accumulation and associated improvement in cellular, molecular, biochemical and behavioral indices are summarized in Table 2.

Drug	Target	Disease Model	Pre/Clinical	Effect	Study
Gemst	Anti-inflammatory Poly-l-lysine compound	Stroke	Rat model of ischemia	Treatment down-regulates IDO pathway, reduction of microglia activation and gliosis in late phase of stroke	[224]
1-MT	IDO inhibitor	epilepsy-induced depression	Pilocarpine induced Chronic temporal lobe epilepsy in rats	Prevented depressive-like behavior but did not influence spontaneous seizures	[225]
1-MT	IDO inhibitor	Inflammation-induced depression	Mice treated with LPS (ICV)	Treatment prevented depressive-like behavior	[226]
Coptisine	IDO inhibitor	Alzheimer’s Disease	Alzheimer’s transgenic AβPP/PS mice	Prevented neuronal cell loss and cognitive impairment, reduced amyloid plague development; decreased activation of microglia and astrocytes	[227]
*N*-(2-*N*,*N*-dimethylaminoethyl)-4-oxo-1*H*-quinoline-2-carboxamide hydrochloride-SZR-72	KA analogue	Huntington’s Disease	HD N171-82Q transgenic mouse model	Treatment increased survival, restored locomotor activity, increased body weight and prevented striatum neuronal loss	[228]
2-(2-*N*,*N*-dimethylaminoethylamine-1carbonyl)-1*H*-quinolin-4-one hydrochloride	KA analogue	Stroke	Rat model of global forebrain ischemia	Treatment prevents neuronal cell loss in CA1 region; preserves induction of LTP	[229]
SZR104	KA analogue	Epilepsy	Pentylenetetrazole induced epileptiform seizures in rats	Treatment decreased population spike activity and epileptiform seizures	[230]
Ro-61-8048	KMO inhibitor	Parkinson’s	MPTP cynomolgus monkey	Chronic treatment with Ro-61-8048 reduced levodopa-induced dyskinesia’s	[228]
Ro-61-8048	KMO inhibitor	Paroxysmal dystonia	dt^sz^ mutant Syrian golden hamsters	Anti-dystonic; reduced severity of dystonic attacks	[231]
Ro-61-8048	KMO inhibitor	Brain injury/Neurotrauma	Surgical brain injury in rats	Treatment reduced brain edema and improved long-term neurological function after SBI	[232]
Ro 61-8048	KMO inhibitor	Spared nerve injury model	Neuropathic pain and depression like behavior in SNI mice	Anti-depressant effect following treatment but no effect on improving mechanical allodynia	[233]
Ro-61-8048, mNBA	KMO inhibitor	Stroke	Rat and gerbil models of ischemia	Treatment prevented neuronal loss and decreased infarct volume	[234]
JM6	KMO inhibitor	Alzheimer’s and Huntington’s	AD (APPtg) and HD (R6/2) transgenic mouse models	Prevents AD and HD behavioral deficits and synaptic loss; increased life span of HD mice	[235]
CHDI-340246	KMO inhibitor	Huntington’s Disease	Multiple HD transgenic mouse models	Treatment ameliorates electrophysiological alterations in striatum, no improvement in behavioral phenotype	[236]
PNU 156561A	KMO inhibitor	Huntington’s Disease	Chemical model of HD in rat; QA induced neurodegeneration and seizures	Anti-convulsant and neuroprotective	[237]
Leucine	LAT1 competitor	Inflammation-induced depression	LPS induced depressive like behavior	Anti-depressant effect due to blockade of kynurenine entry to the brain.	[238]
Electroconvulsive therapy	NA	Depression	Humans with MDD	Treatment improved depressive symptoms; reduced kyn and QA levels in plasma, significantly reduced the QA/KA ratio	[239]
Electroconvulsive therapy	NA	Depression	Humans with MDD	Treatment improved depressive symptoms, increased serum KA levels, increased the KYN/TRP, KA/KYN, and KA/3-HK ratios	[240]
Ketamine	NMDAR antagonist	Inflammation-induced depression; Treatment Resistant Depression	Mice treated with LPS; humans with TRD	Reduces QA and elevates KA in mice treated with LPS, reduced depressive behaviors	[34]
Ketamine	NMDAR antagonist	Treatment Resistant Depression	Humans with TRD	Treatment reduced depressive symptoms; elevated KYNA and KA/KYN ratio in responders	[241]
Ketamine	NMDAR antagonist	Treatment Resistant Depression	Humans with TRD	Treatment reduced depressive symptoms; statistical non-significant trend of elevating KA and KA/KYN	[242]
AV-101 (4-Chloro-kynurenine)	selective GlyB-NMDA receptor antagonist	Epilepsy	Kainate induced seizures and lesion	Anti-convulsant and neuroprotective effects, prevents seizures and lesion in rats	[243]
AV-101 (4-Chloro-kynurenine)	selective GlyB-NMDA receptor antagonist	Depression	Healthy C57BL/6J mice	Anti-depressant like effects on behavioral paradigms used in mice	[244]
AV-101 (4-Chloro-kynurenine)	selective GlyB-NMDA receptor antagonist	Pain	Capsaicin induced experimental pain in humans	Treatment trends to decrease in algesic response, feeling of wellbeing in small cohort of patients	[245]
Escitalopram	SSRI	Depression	Humans with MDD	Treatment improved depressive symptoms, reduced QA and 3-HK, increased KA/QA ratio	[246]
Escitalopram + celecoxib	SSRI, COX-2 inhibitor	Bipolar disorder	Treatment resistant Bipolar depression	Combination of drugs improved treatment response, statistically non-significant trend of reduced peripheral QA	[247]
amitriptyline, imipramine, fluoxetine, citalopram	SSRI, TCAs	Depression	Humans with MDD	All antidepressants increase KA level in hippocampus and increased KAT1 and KAT2 expression in hippocampus	[248]
D-1MT, allopurinol, Ro 61-8048	IDO inhibitor, TDO inhibitor, KMO inhibitor	Schizophrenia	Ketamine-induced increased locomotor activity in rats	All inhibitors reverse the increased locomotor activity in response to ketamine	[249]
Allopurinol	TDO inhibitor	Depression	Rat model of chronic restraint stress	Reduced immobility time in forced swim test, prevented increase in kynurenine levels induced by stress	[250]

**Table 3:** Single Nucleotide Polymorphisms found in the Enzymes involved in KP metabolism associated with CNS diseases. Recent genome wide studies have discovered SNP’s on KP metabolism genes that co-relate with patient symptom severity and treatment outcome. These critical associations will aid in understanding genetic risk, susceptibility and vulnerabilities factors in conjunction to developing animal models, tools and novel therapeutic strategies to target KP metabolism for CNS diseases.

Gene Allele	SNP	Disease Model	Nature of Association with SNP	Study
*TDO2*	rs3755910	Autism	Polymorphism in TDO2 promoter region; CC genotype associated with autism	[286]
*KMO*	rs1053230	Bipolar disorder	Associated with elevated CSF KA in bipolar patients and common in bipolar patients with psychotic features during manic episode	[274]
*KMO*	rs1053230, rs2275163	Depression	No association between rs2275163 and depression. Association trend for rs 1053230 G/G genotype and depression	[276]
*IDO2*	rs2929115, rs2929116	Depression	Nature of association unclear, SNP may alter SSRI citalopram response	[279]
*KATII*, *KMO*	*KATII*-rs1480544, *KMO*-rs1053230	Depression in HIV patients	C-allele in KATII-rs1480544 appears to be protective, KATII-TT-carriers at risk	[277]
*IDO1*	rs9657182	Inflammation-induced depression	Associated with depressive symptoms development post immune therapy; CC genotype more at risk that TT genotype	[287]
*KAT III*, *KMO*	*KAT III*-rs12729558, *KMO*-rs1053230	Major Depressive Disorder and Bipolar Disorder	Associated with bipolar disorder and trend for association with MDD	[278]
*ACMSD*	p.Trp26Stop codon	Familial cortical myoclonic tremor and epilepsy	ACMSD p.Trp26Stop mutation associated with family history of FCMTE	[283]
*ACMSD*	rs775129424	Parkinson’s Disease	Risk associated mutation found in patient with PD; affects response to treatment	[284]
*ACMSD*	rs6710823	Parkinson’s Disease	Meta-analysis of GWAS reveals ACMSD SNP predicts PD risk	[285]
*KMO*	rs1053230	Post-partum depression	Associated with elevated levels of 3-HK and higher 3-HK/KYN ratio in serum of women in post-partum depression. Significantly associated with post-partum depression	[280]
*IDO1*	rs10108662	Post-partum depression	Associated with post-partum depression	[281]
*KATI*, *KATII*, *KATIII*	NA	Post-partum depression	No association between eight KAT I–III SNPs and PPD	[282]
*KMO*	rs1053230	Schizophrenia	SNP associated with Increased KA in the CSF of schizophrenia patient	[288]
*KMO*	rs1053230, rs2275163	Schizophrenia	Increased risk were the GG genotype (rs1053230) and the T*GG diplotype (rs2275163 and rs1053230)	[289]
*KMO*	rs2275163	Schizophrenia	Associated with oculomotor measures of predictive pursuit and visuospatial working memory deficits in schizophrenic patients	[272]
*KMO*	rs2275163, rs1053230	Schizophrenia	rs2275163C>T allele is associated with poor performance on battery of cognitive tests in schizophrenia with decrease cognitive scores associated but rs1053230 is not	[273]
*KMO*	rs2275163	Schizophrenia	Associated with schizophrenia in one cohort, but effect was not replicated in second larger cohort	[275]
*ACMSD*	rs2121337	Suicide	SNP associated with higher QA in suicidal patients and reduced PA production	[154]

## Data Availability

Not applicable.

## Abbreviations

1-MT	1-methyltryptophan
3-HAAO	3-hydoxyanthranillic acid dioxygenase
3-HK	3-Hydroxykynurenine
α7nAChR	Alpha-7-nicotinic acetylcholine receptor
AβPP/PS	double transgenic mice expressing a chimeric mouse/human amyloid precursor protein (Mo/HuAPP695swe) and a mutant human presenilin 1 (PS1-dE9)
ACMSD	2-Amino-3-carboxymuconic-6-semiladehyde decarboxylase
AD	Alzheimer’s disease
AhR	Aryl hydrocarbon receptor
BBB	blood–brain barrier
CA	Cinnabarinic acid
CA1	Cornu Ammonis 1
CNS	Central nervous system
COX2	Cyclooxygenase 2
CSF	Cerebrospinal fluid
FCMTE	Familial cortical myoclonic tremor with epilepsy
GlyB	Glycine B Site
GPR35	G-protein couples receptor 35
GWAS	Genome Wide Association Studies
HD	Huntington’s disease
HIV	Human immunodeficiency virus
ICV	Intracerebroventricular
IDO	Indoleamine-2,3-dioxygenase
KAT	Kynurenine aminotransferase
KA	Kynurenic acid
KAT	Kynurenine aminotransferase
Kyn	Kynurenine
KMO	Kynurenine-3-monooxygenase
KP	kynurenine Pathway
LAT1	Large amino acid transporter 1
LPS	Lipopolysaccharide
LTP	Long term potentiation
MDD	Major depressive disorder
mGLUR	Metabotropic glutamate receptors
MPTP	1-Methyl-4-phenyl-1,2,3,6-tetrahydropyridine
NAD	Nicotinamide adenine dinucleotide
NMDAR	N-methyl-D-aspartate receptor
PD	Parkinson’s disease
PA	Picolinic acid
PDD	Postpartum depression
QA	Quinolinic acid
SBI	Surgical brain injury
SNI	Spared nerve injury
SNP	Single nucleotide polymorphism
SNRI	Selective norepinephrine reuptake inhibitor
SSRI	Selective serotonin reuptake inhibitor
TCA	Tricyclic antidepressants
TDO	Tryptophan-2,3-dioxygnease
TRD	Treatment resistant depression
TRP	Tryptophan
VGLUT	Vesicle glutamate transporter

## References

[1] Feigin V.L., Krishnamurthi R.V., Theadom A.M., Abajobir A.A., Mishra S.R., Ahmed M.B., Abate K.H., Mengistie M.A., Wakayo T., Abd-Allah F. (2017). Global, regional, and national burden of neurological disorders during 1990–2015: A systematic analysis for the Global Burden of Disease Study 2015. Lancet Neurol..

[2] Gooch C.L., Pracht E., Borenstein A.R. (2017). The burden of neurological disease in the United States: A summary report and call to action. Ann. Neurol..

[3] Jayaraj R.L., Azimullah S., Beiram R., Jalal F.Y., Rosenberg G.A. (2019). Neuroinflammation: Friend and foe for ischemic stroke. J. Neuroinflammation.

[4] Calsolaro V., Edison P. (2016). Neuroinflammation in Alzheimer’s disease: Current evidence and future directions. Alzheimer’s Dement..

[5] Fourrier C., Singhal G., Baune B.T. (2019). Neuroinflammation and cognition across psychiatric conditions. CNS Spectr..

[6] Lin S.-J., Guarente L. (2003). Nicotinamide adenine dinucleotide, a metabolic regulator of transcription, longevity and disease. Curr. Opin. Cell Biol..

[7] Guzman-Martinez L., Maccioni R.B., Andrade V., Navarrete L.P., Pastor M.G., Ramos-Escobar N. (2019). Neuroinflammation as a common feature of neurodegenerative disorders. Front. Pharmacol..

[8] Vogels T., Murgoci A.N., Hromádka T. (2019). Intersection of pathological tau and microglia at the synapse. Acta Neuropathol. Commun..

[9] Spangenberg E.E., Lee R.J., Najafi A.R., Rice R.A., Elmore M.R.P., Blurton-Jones M., West B.L., Green K.N. (2016). Eliminating microglia in Alzheimer’s mice prevents neuronal loss without modulating amyloid-β pathology. Brain.

[10] Spangenberg E., Severson P.L., Hohsfield L.A., Crapser J., Zhang J., Burton E.A., Zhang Y., Spevak W., Lin J., Phan N.Y. (2019). Sustained microglial depletion with CSF1R inhibitor impairs parenchymal plaque development in an Alzheimer’s disease model. Nat. Commun..

[11] Zhu K., Pieber M., Han J., Blomgren K., Zhang X.M., Harris R.A., Lund H. (2020). Absence of microglia or presence of peripherally-derived macrophages does not affect tau pathology in young or old hTau mice. Glia.

[12] Gosselin D., Skola D., Coufal N.G., Holtman I.R., Schlachetzki J.C.M., Sajti E., Jaeger B.N., O’Connor C., Fitzpatrick C., Pasillas M.P. (2017). An environment-dependent transcriptional network specifies human microglia identity. Science.

[13] Galatro T.F., Holtman I.R., Lerario A.M., Vainchtein I.D., Brouwer N., Sola P.R., Veras M.M., Pereira T.F., Leite R.E.P., Möller T. (2017). Transcriptomic analysis of purified human cortical microglia reveals age-associated changes. Nat. Neurosci..

[14] Nation D.A., Sweeney M.D., Montagne A., Sagare A.P., D’Orazio L.M., Pachicano M., Sepehrband F., Nelson A.R., Buennagel D.P., Harrington M.G. (2019). Blood–brain barrier breakdown is an early biomarker of human cognitive dysfunction. Nat. Med..

[15] Swardfager W., Lanctôt K., Rothenburg L., Wong A., Cappell J., Herrmann N. (2010). A Meta-Analysis of Cytokines in Alzheimer’s Disease. Biol. Psychiatry.

[16] Newcombe E.A., Camats-Perna J., Silva M.L., Valmas N., Huat T.J., Medeiros R. (2018). Inflammation: The link between comorbidities, genetics, and Alzheimer’s disease. J. Neuroinflammation.

[17] Chitnis T., Weiner H.L. (2017). Series Editors : Marco Colonna and David Holtzmann CNS inflammation and neurodegeneration. J. Clin. Invest..

[18] Wyss-Coray T., Mucke L. (2002). Inflammation in Neurodegenerative Disease—A Double-Edged Sword. Neuron.

[19] Solano Fonseca R., Mahesula S., Apple D.M., Raghunathan R., Dugan A., Cardona A., O’Connor J., Kokovay E. (2016). Neurogenic niche microglia undergo positional remodeling and progressive activation contributing to age-associated reductions in neurogenesis. Stem Cells Dev..

[20] Jurgens H.A., Amancherla K., Johnson R.W. (2012). Influenza Infection Induces Neuroinflammation, Alters Hippocampal Neuron Morphology, and Impairs Cognition in Adult Mice. J. Neurosci..

[21] Haroon E., Raison C.L., Miller A.H. (2012). Psychoneuroimmunology meets neuropsychopharmacology: Translational implications of the impact of inflammation on behavior. Neuropsychopharmacology.

[22] Singhal G., Baune B.T. (2020). Inflammatory Abnormalities in Major Depressive Disorder. Major Depressive Disorder.

[23] Capuron L., Gumnick J.F., Musselman D.L., Lawson D.H., Reemsnyder A., Nemeroff C.B., Miller A.H. (2002). Neurobehavioral effects of interferon-α in cancer patients: Phenomenology and paroxetine responsiveness of symptom dimensions. Neuropsychopharmacology.

[24] Larkin P.B., Sathyasaikumar K.V., Notarangelo F.M., Funakoshi H., Nakamura T., Schwarcz R., Muchowski P.J. (2016). Tryptophan 2,3-dioxygenase and indoleamine 2,3-dioxygenase 1 make separate, tissue-specific contributions to basal and inflammation-induced kynurenine pathway metabolism in mice. Biochim. Biophys. Acta Gen. Subj..

[25] Laugeray A., Launay J.M., Callebert J., Mutlu O., Guillemin G.J., Belzung C., Barone P.R. (2016). Chronic treatment with the IDO1 inhibitor 1-methyl-D-tryptophan minimizes the behavioural and biochemical abnormalities induced by unpredictable chronic mild stress in mice—Comparison with fluoxetine. PLoS ONE.

[26] Meyer P.F., Tremblay-Mercier J., Leoutsakos J., Madjar C., Lafaille-Maignan M.É., Savard M., Rosa-Neto P., Poirier J., Etienne P., Breitner J. (2019). A randomized trial of naproxen to slow progress of presymptomatic Alzheimer disease. Neurology.

[27] Aisen P.S., Schafer K.A., Grundman M., Pfeiffer E., Sano M., Davis K.L., Farlow M.R., Jin S., Thomas R.G., Thal L.J. (2003). Effects of Rofecoxib or Naproxen vs Placebo on Alzheimer Disease Progression: A Randomized Controlled Trial. J. Am. Med. Assoc..

[28] Butchart J., Brook L., Hopkins V., Teeling J., Püntener U., Culliford D., Sharples R., Sharif S., McFarlane B., Raybould R. (2015). Etanercept in Alzheimer disease. Neurology.

[29] Drieu A., Levard D., Vivien D., Rubio M. (2018). Anti-inflammatory treatments for stroke: From bench to bedside. Ther. Adv. Neurol. Disord..

[30] Lazar M.A., McIntyre R.S. (2019). Novel Therapeutic Targets for Major Depressive Disorder. Neurobiology of Depression.

[31] Nettis M.A., Lombardo G., Hastings C., Zajkowska Z., Mariani N., Nikkheslat N., Worrell C., Enache D., McLaughlin A., Kose M. (2021). Augmentation therapy with minocycline in treatment-resistant depression patients with low-grade peripheral inflammation: Results from a double-blind randomised clinical trial. Neuropsychopharmacology.

[32] Husain M.I., Cullen C., Umer M., Carvalho A.F., Kloiber S., Meyer J.H., Ortiz A., Knyahnytska Y., Husain M.O., Giddens J. (2020). Minocycline as adjunctive treatment for treatment-resistant depression: Study protocol for a double blind, placebo-controlled, randomized trial (MINDEP2). BMC Psychiatry.

[33] Rosenblat J.D., McIntyre R.S. (2018). Efficacy and tolerability of minocycline for depression: A systematic review and meta-analysis of clinical trials. J. Affect. Disord..

[34] Verdonk F., Petit A.C., Abdel-Ahad P., Vinckier F., Jouvion G., de Maricourt P., De Medeiros G.F., Danckaert A., Van Steenwinckel J., Blatzer M. (2019). Microglial production of quinolinic acid as a target and a biomarker of the antidepressant effect of ketamine. Brain. Behav. Immun..

[35] Bhattacharya A., Lord B., Grigoleit J.S., He Y., Fraser I., Campbell S.N., Taylor N., Aluisio L., O’Connor J.C., Papp M. (2018). Neuropsychopharmacology of JNJ-55308942: Evaluation of a clinical candidate targeting P2X7 ion channels in animal models of neuroinflammation and anhedonia. Neuropsychopharmacology.

[36] Mallah K., Couch C., Borucki D.M., Toutonji A., Alshareef M., Tomlinson S. (2020). Anti-inflammatory and Neuroprotective Agents in Clinical Trials for CNS Disease and Injury: Where Do We Go From Here?. Front. Immunol..

[37] Colpo G.D., Venna V.R., McCullough L.D., Teixeira A.L. (2019). Systematic review on the involvement of the kynurenine pathway in stroke: Pre-clinical and Clinical Evidence. Front. Neurol..

[38] Howard R., Zubko O., Bradley R., Harper E., Pank L., O’Brien J., Fox C., Tabet N., Livingston G., Bentham P. (2020). Minocycline at 2 Different Dosages vs Placebo for Patients with Mild Alzheimer Disease: A Randomized Clinical Trial. JAMA Neurol..

[39] Parashos S.A., Luo S., Biglan K.M., Bodis-Wollner I., Liang G.S., Ross G.W., Tilley B.C., Shulman L.M. (2014). Measuring disease progression in early parkinson disease the national institutes of health exploratory trials in parkinson disease (NET-PD) experience. JAMA Neurol..

[40] Henry R.J., Ritzel R.M., Barrett J.P., Doran S.J., Jiao Y., Leach J.B., Szeto G.L., Wu J., Stoica B.A., Faden A.I. (2020). Microglial depletion with CSF1R inhibitor during chronic phase of experimental traumatic brain injury reduces neurodegeneration and neurological deficits. J. Neurosci..

[41] Li X., Redus L., Chen C., Martinez P.A., Strong R., Li S., O’Connor J.C. (2013). Cognitive Dysfunction Precedes the Onset of Motor Symptoms in the MitoPark Mouse Model of Parkinson’s Disease. PLoS ONE.

[42] Katsyuba E., Romani M., Hofer D., Auwerx J. (2020). NAD+ homeostasis in health and disease. Nat. Metab..

[43] Stone T.W., Forrest C.M., Darlington L.G. (2012). Kynurenine pathway inhibition as a therapeutic strategy for neuroprotection. FEBS J..

[44] Ball H.J., Yuasa H.J., Austin C.J.D., Weiser S., Hunt N.H. (2009). Indoleamine 2,3-dioxygenase-2; a new enzyme in the kynurenine pathway. Int. J. Biochem. Cell Biol..

[45] Han Q., Robinson H., Li J. (2012). Biochemical identification and crystal structure of kynurenine formamidase from Drosophila melanogaster. Biochem. J..

[46] Lugo-Huitron R., Ugalde Muniz P., Pineda B., Pedraza-Chaverri J., Rios C., Perez-De La Cruz V. (2013). Quinolinic acid: An endogenous neurotoxin with multiple targets. Oxid. Med. Cell. Longev..

[47] Baumgartner R., Forteza M.J., Ketelhuth D.F.J. (2019). The interplay between cytokines and the Kynurenine pathway in inflammation and atherosclerosis. Cytokine.

[48] Stone T.W., Darlington L.G. (2002). Endogenous kynurenines as targets for drug discovery and development. Nat. Rev. Drug Discov..

[49] Campbell B.M., Charych E., Lee A.W., Möller T. (2014). Kynurenines in CNS disease: Regulation by inflammatory cytokines. Front. Neurosci..

[50] Lawson M.A., Parrott J.M., McCusker R.H., Dantzer R., Kelley K.W., O’Connor J.C. (2013). Intracerebroventricular administration of lipopolysaccharide induces indoleamine-2,3-dioxygenase-dependent depression-like behaviors. J. Neuroinflammation.

[51] O’Connor J.C., Lawson M.A., André C., Briley E.M., Szegedi S.S., Lestage J., Castanon N., Herkenham M., Dantzer R., Kelley K.W. (2009). Induction of IDO by Bacille Calmette-Guérin Is Responsible for Development of Murine Depressive-Like Behavior. J. Immunol..

[52] O’Connor J.C., Lawson M.A., André C., Moreau M., Lestage J., Castanon N., Kelley K.W., Dantzer R. (2008). Lipopolysaccharide-induced depressive-like behavior is mediated by indoleamine 2,3-dioxygenase activation in mice. Mol. Psychiatry.

[53] Schwarcz R., Bruno J.P., Muchowski P.J., Wu H.Q. (2012). Kynurenines in the mammalian brain: When physiology meets pathology. Nat. Rev. Neurosci..

[54] Heyes M.P., Achim C.L., Wiley C.A., Major E.O., Saito K., Markey S.P. (1996). Human microglia convert L-tryptophan into the neurotoxin quinolinic acid. Biochem. J..

[55] Guillemin G.J., Kerr S.J., Smythe G.A., Smith D.G., Kapoor V., Armati P.J., Croitoru J., Brew B.J. (2001). Kynurenine pathway metabolism in human astrocytes: A paradox for neuronal protection. J. Neurochem..

[56] Schwarcz R., Whetsell W., Mangano R. (1983). Quinolinic acid: An endogenous metabolite that produces axon-sparing lesions in rat brain. Science.

[57] Foster A.C., White R.J., Schwarcz R. (1986). Synthesis of Quinolinic Acid by 3-Hydroxyanthranilic Acid Oxygenase in Rat Brain Tissue In Vitro. J. Neurochem..

[58] Foster A.C., Zinkand W.C., Schwarcz R. (1985). Quinolinic Acid Phosphoribosyltransferase in Rat Brain. J. Neurochem..

[59] Pucci L., Perozzi S., Cimadamore F., Orsomando G., Raffaelli N. (2007). Tissue expression and biochemical characterization of human 2-amino 3-carboxymuconate 6-semialdehyde decarboxylase, a key enzyme in tryptophan catabolism. FEBS J..

[60] Han Q., Cai T., Tagle D.A., Li J. (2010). Structure, expression, and function of kynurenine aminotransferases in human and rodent brains. Cell. Mol. Life Sci..

[61] Guidetti P., Hoffman G.E., Melendez-Ferro M., Albuquerque E.X., Schwarcz R. (2007). Astrocytic localization of kynurenine aminotransferase II in the rat brain visualized by immunocytochemistry. Glia.

[62] Baran H., Schwarcz R. (1990). Presence of 3-Hydroxyanthranilic Acid in Rat Tissues and Evidence for Its Production from Anthranilic Acid in the Brain. J. Neurochem..

[63] Fazio F., Lionetto L., Curto M., Iacovelli L., Cavallari M., Zappulla C., Ulivieri M., Napoletano F., Capi M., Corigliano V. (2015). Xanthurenic Acid Activates mGlu2/3 metabotropic glutamate receptors and is a potential trait marker for schizophrenia. Sci. Rep..

[64] Sathyasaikumar K.V., Tararina M., Wu H.-Q., Neale S.A., Weisz F., Salt T.E., Schwarcz R. (2017). Xanthurenic Acid Formation from 3-Hydroxykynurenine in the Mammalian Brain: Neurochemical Characterization and Physiological Effects. Neuroscience.

[65] Fazio F., Lionetto L., Molinaro G., Bertrand H.O., Acher F., Ngomba R.T., Notartomaso S., Curini M., Rosati O., Scarselli P. (2012). Cinnabarinic acid, an endogenous metabolite of the kynurenine pathway, activates type 4 metabotropic glutamate receptors. Mol. Pharmacol..

[66] Fukui S., Schwarcz R., Rapoport S.I., Takada Y., Smith Q.R. (1991). Blood–Brain Barrier Transport of Kynurenines: Implications for Brain Synthesis and Metabolism. J. Neurochem..

[67] Guillemin G.J., Cullen K.M., Lim C.K., Smythe G.A., Garner B., Kapoor V., Takikawa O., Brew B.J. (2007). Characterization of the kynurenine pathway in human neurons. J. Neurosci..

[68] Strasser B., Becker K., Fuchs D., Gostner J.M. (2017). Kynurenine pathway metabolism and immune activation: Peripheral measurements in psychiatric and co-morbid conditions. Neuropharmacology.

[69] Bradley K.A.L., Case J.A.C., Khan O., Ricart T., Hanna A., Alonso C.M., Gabbay V. (2015). The role of the kynurenine pathway in suicidality in adolescent major depressive disorder. Psychiatry Res..

[70] Yoshida R., Imanishi J., Oku T., Kishida T., Hayaishi O. (1981). Induction of pulmonary indoleamine 2,3-dioxygenase by interferon. Proc. Natl. Acad. Sci. USA.

[71] Musso T., Gusella G.L., Brooks A., Longo D.L., Varesio L. (1994). Interleukin-4 inhibits indoleamine 2,3-dioxygenase expression in human monocytes. Blood.

[72] Connor J.C.O., Andre C., Wang Y., Lawson M.A., Szegedi S.S., Lestage J., Castanon N., Kelley K.W., Dantzer R. (2009). Interferon-y and Tumor Necrosis Factor-Mediate the Upregulation of Indoleamine 2,3-Dioxygenase and the Induction of Depressive-Like Behavior in Mice in Response to Bacillus Calmette-Guerin. J. Neurosci..

[73] Huang Y.S., Ogbechi J., Clanchy F.I., Williams R.O., Stone T.W. (2020). IDO and Kynurenine Metabolites in Peripheral and CNS Disorders. Front. Immunol..

[74] Gál E.M., Sherman A.D. (1980). L-kynurenine: Its synthesis and possible regulatory function in brain. Neurochem. Res..

[75] Abdullahi W., Tripathi D., Ronaldson P.T. (2018). Blood-brain barrier dysfunction in ischemic stroke: Targeting tight junctions and transporters for vascular protection. Am. J. Physiol.-Cell Physiol..

[76] Parrott J.M., O’Connor J.C. (2015). Kynurenine 3-monooxygenase: An influential mediator of neuropathology. Front. Psychiatry.

[77] Farzi A., Reichmann F., Meinitzer A., Mayerhofer R., Jain P., Hassan A.M., Fröhlich E.E., Wagner K., Painsipp E., Rinner B. (2015). Synergistic effects of NOD1 or NOD2 and TLR4 activation on mouse sickness behavior in relation to immune and brain activity markers. Brain. Behav. Immun..

[78] Wohleb E.S., Powell N.D., Godbout J.P., Sheridan J.F. (2013). Stress-induced recruitment of bone marrow-derived monocytes to the brain promotes anxiety-like behavior. J. Neurosci..

[79] Majerova P., Michalicova A., Cente M., Hanes J., Vegh J., Kittel A., Kosikova N., Cigankova V., Mihaljevic S., Jadhav S. (2019). Trafficking of immune cells across the bloodbrain barrier is modulated by neurofibrillary pathology in tauopathies. PLoS ONE.

[80] Owe-Young R., Webster N.L., Mukhtar M., Pomerantz R.J., Smythe G., Walker D., Armati P.J., Crowe S.M., Brew B.J. (2008). Kynurenine pathway metabolism in human blood-brain-barrier cells: Implications for immune tolerance & neurotoxicity. J. Neurochem..

[81] Däubener W., Spors B., Hucke C., Adam R., Stins M., Kim K.S., Schroten H. (2001). Restriction of Toxoplasma gondii growth in human brain microvascular endothelial cells by activation of indoleamine 2,3-dioxygenase. Infect. Immun..

[82] Adam R., Russing D., Adams O., Ailyati A., Sik Kim K., Schroten H., Daubener W. (2005). Role of human brain microvascular endothelial cells during central nervous system infection. Significance of indoleamine 2,3-dioxygenase in antimicrobial defence and immunoregulation. Thromb. Haemost..

[83] Liu H., Liu L., Liu K., Bizargity P., Hancock W.W., Visner G.A. (2009). Reduced Cytotoxic Function of Effector CD^8+^ T Cells Is Responsible for Indoleamine 2,3-Dioxygenase-Dependent Immune Suppression. J. Immunol..

[84] Donley D.W., Olson A.R., Raisbeck M.F., Fox J.H., Gigley J.P. (2016). Huntingtons disease mice infected with toxoplasma gondii demonstrate early kynurenine pathway activation, altered CD8+ T-Cell responses, and premature mortality. PLoS ONE.

[85] Zang X., Zheng X., Hou Y., Hu M., Wang H., Bao X., Zhou F., Wang G., Hao H. (2018). Regulation of proinflammatory monocyte activation by the kynurenine-AhR axis underlies immunometabolic control of depressive behavior in mice. FASEB J..

[86] Darlington L.G., Mackay G.M., Forrest C.M., Stoy N., George C., Stone T.W. (2007). Altered kynurenine metabolism correlates with infarct volume in stroke. Eur. J. Neurosci..

[87] Garrison A.M., Parrott J.M., Tuñon A., Delgado J., Redus L., O’Connor J.C. (2018). Kynurenine pathway metabolic balance influences microglia activity: Targeting kynurenine monooxygenase to dampen neuroinflammation. Psychoneuroendocrinology.

[88] Carlessi A.S., Borba L.A., Zugno A.I., Quevedo J., Réus G.Z. (2019). Gut microbiota–brain axis in depression: The role of neuroinflammation. Eur. J. Neurosci..

[89] Gao K., Mu C., Farzi A., Zhu W. (2019). Tryptophan Metabolism: A Link Between the Gut Microbiota and Brain. Adv. Nutr..

[90] Dehhaghi M., Kazemi Shariat Panahi H., Guillemin G.J. (2019). Microorganisms, Tryptophan Metabolism, and Kynurenine Pathway: A Complex Interconnected Loop Influencing Human Health Status. Int. J. Tryptophan Res..

[91] Luczynski P., Whelan S.O., O’Sullivan C., Clarke G., Shanahan F., Dinan T.G., Cryan J.F. (2016). Adult microbiota-deficient mice have distinct dendritic morphological changes: Differential effects in the amygdala and hippocampus. Eur. J. Neurosci..

[92] Gao J., Xu K., Liu H., Liu G., Bai M., Peng C., Li T., Yin Y. (2018). Impact of the gut microbiota on intestinal immunity mediated by tryptophan metabolism. Front. Cell. Infect. Microbiol..

[93] Kennedy P.J., Cryan J.F., Dinan T.G., Clarke G. (2017). Kynurenine pathway metabolism and the microbiota-gut-brain axis. Neuropharmacology.

[94] Cryan J.F., Dinan T.G. (2012). Mind-altering microorganisms: The impact of the gut microbiota on brain and behaviour. Nat. Rev. Neurosci..

[95] Bercik P., Verdu E.F., Foster J.A., MacRi J., Potter M., Huang X., Malinowski P., Jackson W., Blennerhassett P., Neufeld K.A. (2010). Chronic gastrointestinal inflammation induces anxiety-like behavior and alters central nervous system biochemistry in mice. Gastroenterology.

[96] Bailey M.T., Dowd S.E., Galley J.D., Hufnagle A.R., Allen R.G., Lyte M. (2011). Exposure to a social stressor alters the structure of the intestinal microbiota: Implications for stressor-induced immunomodulation. Brain. Behav. Immun..

[97] Parrott J.M., Redus L., Santana-Coelho D., Morales J., Gao X., O’Connor J.C. (2016). Neurotoxic kynurenine metabolism is increased in the dorsal hippocampus and drives distinct depressive behaviors during inflammation. Transl. Psychiatry.

[98] Parrott J.M., Redus L., Connor J.C.O. (2016). Kynurenine metabolic balance is disrupted in the hippocampus following peripheral lipopolysaccharide challenge. J. Neuroinflammation.

[99] Heyes M.P., Nowak T.S. (1990). Delayed increases in regional brain quinolinic acid follow transient ischemia in the gerbil. J. Cereb. Blood Flow Metab..

[100] Koo Y.S., Kim H., Park J.H., Kim M.J., Shin Y.I., Choi B.T., Lee S.Y., Shin H.K. (2018). Indoleamine 2,3-dioxygenase-dependent neurotoxic kynurenine metabolism contributes to poststroke depression induced in mice by ischemic stroke along with spatial restraint stress. Oxid. Med. Cell. Longev..

[101] Bosnyák E., Kamson D.O., Behen M.E., Barger G.R., Mittal S., Juhász C. (2015). Imaging cerebral tryptophan metabolism in brain tumor-associated depression. EJNMMI Res..

[102] Opitz C.A., Litzenburger U.M., Sahm F., Ott M., Tritschler I., Trump S., Schumacher T., Jestaedt L., Schrenk D., Weller M. (2011). An endogenous tumour-promoting ligand of the human aryl hydrocarbon receptor. Nature.

[103] Cuartero M.I., Ballesteros I., De La Parra J., Harkin A.L., Abautret-Daly A., Sherwin E., Fernández-Salguero P., Corbí Á.L., Lizasoain I., Moro M.A. (2014). L-kynurenine/aryl hydrocarbon receptor pathway mediates brain damage after experimental stroke. Circulation.

[104] Kondrikov D., Elmansi A., Bragg R.T., Mobley T., Barrett T., Eisa N., Kondrikova G., Schoeinlein P., Aguilar-Perez A., Shi X.M. (2020). Kynurenine inhibits autophagy and promotes senescence in aged bone marrow mesenchymal stem cells through the aryl hydrocarbon receptor pathway. Exp. Gerontol..

[105] García-Lara L., Pérez-Severiano F., González-Esquivel D., Elizondo G., Segovia J. (2015). Absence of aryl hydrocarbon receptors increases endogenous kynurenic acid levels and protects mouse brain against excitotoxic insult and oxidative stress. J. Neurosci. Res..

[106] Moyer B.J., Rojas I.Y., Kerley-Hamilton J.S., Hazlett H.F., Nemani K.V., Trask H.W., West R.J., Lupien L.E., Collins A.J., Ringelberg C.S. (2016). Inhibition of the aryl hydrocarbon receptor prevents Western diet-induced obesity. Model for AHR activation by kynurenine via oxidized-LDL, TLR2/4, TGFβ, and IDO1. Toxicol. Appl. Pharmacol..

[107] Du L., Xing Z., Tao B., Li T., Yang D., Li W., Zheng Y., Kuang C., Yang Q. (2020). Both IDO1 and TDO contribute to the malignancy of gliomas via the Kyn-AhR-AQP4 signaling pathway. Signal Transduct. Target. Ther..

[108] Manni G., Mondanelli G., Scalisi G., Pallotta M.T., Nardi D., Padiglioni E., Romani R., Talesa V.N., Puccetti P., Fallarino F. (2020). Pharmacologic Induction of Endotoxin Tolerance in Dendritic Cells by L-Kynurenine. Front. Immunol..

[109] Li Q., Harden J.L., Anderson C.D., Egilmez N.K. (2016). Tolerogenic Phenotype of IFN-γ–Induced IDO + Dendritic Cells Is Maintained via an Autocrine IDO–Kynurenine/AhR–IDO Loop. J. Immunol..

[110] Mondanelli G., Coletti A., Greco F.A., Pallotta M.T., Orabona C., Iacono A., Belladonna M.L., Albini E., Panfili E., Fallarino F. (2020). Positive allosteric modulation of indoleamine 2,3-dioxygenase 1 restrains neuroinflammation. Proc. Natl. Acad. Sci. USA.

[111] Yamamoto T., Hatabayashi K., Arita M., Yajima N., Takenaka C., Suzuki T., Takahashi M., Oshima Y., Hara K., Kagawa K. (2019). Kynurenine signaling through the aryl hydrocarbon receptor maintains the undifferentiated state of human embryonic stem cells. Sci. Signal..

[112] Reyes Ocampo J., Lugo Huitrón R., González-Esquivel D., Ugalde-Muñiz P., Jiménez-Anguiano A., Pineda B., Pedraza-Chaverri J., Ríos C., Pérez De La Cruz V. (2014). Kynurenines with neuroactive and redox properties: Relevance to aging and brain diseases. Oxid. Med. Cell. Longev..

[113] Kaiser H., Yu K., Pandya C., Mendhe B., Isales C.M., McGee-Lawrence M.E., Johnson M., Fulzele S., Hamrick M.W. (2019). Kynurenine, a Tryptophan Metabolite That Increases with Age, Induces Muscle Atrophy and Lipid Peroxidation. Oxid. Med. Cell. Longev..

[114] Morita T., Saito K., Takemura M., Maekawa N., Fujigaki S., Fujii H., Wada H., Takeuchi S., Noma A., Seishima M. (2001). 3-Hydroxyanthranilic acid, an L-tryptophan metabolite, induces apoptosis in monocyte-derived cells stimulated by interferon-γ. Ann. Clin. Biochem..

[115] Backhaus C., Rahman H., Scheffler S., Laatsch H., Hardeland R. (2008). NO scavenging by 3-hydroxyanthranilic acid and 3-hydroxykynurenine: N-nitrosation leads via oxadiazoles to o-quinone diazides. Nitric Oxide Biol. Chem..

[116] Song P., Ramprasath T., Wang H., Zou M.H. (2017). Abnormal kynurenine pathway of tryptophan catabolism in cardiovascular diseases. Cell. Mol. Life Sci..

[117] Gargaro M., Vacca C., Massari S., Scalisi G., Manni G., Mondanelli G., Mazza E.M.C., Bicciato S., Pallotta M.T., Orabona C. (2019). Engagement of nuclear coactivator 7 by 3-hydroxyanthranilic acid enhances activation of aryl hydrocarbon receptor in immunoregulatory dendritic cells. Front. Immunol..

[118] Colín-González A.L., Maldonado P.D., Santamaría A. (2013). 3-Hydroxykynurenine: An intriguing molecule exerting dual actions in the Central Nervous System. Neurotoxicology.

[119] Eastman C.L., Guilarte T.R. (1990). The role of hydrogen peroxide in the in vitro cytotoxicity of 3-hydroxykynurenine. Neurochem. Res..

[120] Leipnitz G., Schumacher C., Dalcin K.B., Scussiato K., Solano A., Funchal C., Dutra-Filho C.S., Wyse A.T.S., Wannmacher C.M.D., Latini A. (2007). In vitro evidence for an antioxidant role of 3-hydroxykynurenine and 3-hydroxyanthranilic acid in the brain. Neurochem. Int..

[121] Fazio F., Zappulla C., Notartomaso S., Busceti C., Bessede A., Scarselli P., Vacca C., Gargaro M., Volpi C., Allegrucci M. (2014). Cinnabarinic acid, an endogenous agonist of type-4 metabotropic glutamate receptor, suppresses experimental autoimmune encephalomyelitis in mice. Neuropharmacology.

[122] Fazio F., Lionetto L., Curto M., Iacovelli L., Copeland C.S., Neale S.A., Bruno V., Battaglia G., Salt T.E., Nicoletti F. (2017). Cinnabarinic acid and xanthurenic acid: Two kynurenine metabolites that interact with metabotropic glutamate receptors. Neuropharmacology.

[123] Hilmas C., Pereira E.F.R., Alkondon M., Rassoulpour A., Schwarcz R., Albuquerque E.X. (2001). The brain metabolite kynurenic acid inhibits α7 nicotinic receptor activity and increases non-α7 nicotinic receptor expression: Physiopathological implications. J. Neurosci..

[124] Prescott C., Weeks A.M., Staley K.J., Partin K.M. (2006). Kynurenic acid has a dual action on AMPA receptor responses. Neurosci. Lett..

[125] Lapin I.P. (1983). Antagonism of kynurenine-induced seizures by picolinic, kynurenic and xanthurenic acids. J. Neural Transm..

[126] Ferreira F.S., Biasibetti-Brendler H., Pierozan P., Schmitz F., Bertó C.G., Prezzi C.A., Manfredini V., Wyse A.T.S. (2018). Kynurenic Acid Restores Nrf2 Levels and Prevents Quinolinic Acid-Induced Toxicity in Rat Striatal Slices. Mol. Neurobiol..

[127] Stone T.W. (2020). Does kynurenic acid act on nicotinic receptors? An assessment of the evidence. J. Neurochem..

[128] Fernandez-Pol J.A., Klos D.J., Hamilton P.D. (2001). Antiviral, cytotoxic and apoptotic activities of picolinic acid on human immunodeficiency virus-1 and human herpes simplex virus-2 infected cells. Anticancer Res..

[129] Cai S., Sato K., Shimizu T., Yamabe S., Hiraki M., Sano C., Tomioka H. (2006). Antimicrobial activity of picolinic acid against extracellular and intracellular Mycobacterium avium complex and its combined activity with clarithromycin, rifampicin and fluoroquinolones. J. Antimicrob. Chemother..

[130] Cioczek-Czuczwar A., Czuczwar P., Turski W.A., Parada-Turska J. (2017). Influence of picolinic acid on seizure susceptibility in mice. Pharmacol. Reports.

[131] Santamaría A., Galván-Arzate S., Lisý V., Ali S.F., Duhart H.M., Osorio-Rico L., Ríos C., Sut’astný F. (2001). Quinolinic acid induces oxidative stress in rat brain synaptosomes. Neuroreport.

[132] Neale S.A., Copeland C.S., Salt T.E. (2014). Effect of VGLUT inhibitors on glutamatergic synaptic transmission in the rodent hippocampus and prefrontal cortex. Neurochem. Int..

[133] Neale S.A., Copeland C.S., Uebele V.N., Thomson F.J., Salt T.E. (2013). Modulation of hippocampal synaptic transmission by the kynurenine pathway member xanthurenic acid and other vglut inhibitors. Neuropsychopharmacology.

[134] Kubicova L., Hadacek F., Bachmann G., Weckwerth W., Chobot V. (2019). Coordination complex formation and redox properties of kynurenic and xanthurenic acid can affect brain tissue homeodynamics. Antioxidants.

[135] Speciale C., Schwarcz R. (1990). Uptake of Kynurenine into Rat Brain Slices. J. Neurochem..

[136] Eastman C.L., Guilarte T.R., Lever J.R. (1992). Uptake of 3-hydroxykynurenine measured in rat brain slices and in a neuronal cell line. Brain Res..

[137] Sardar A.M., Bell J.E., Reynolds G.P. (1995). Increased Concentrations of the Neurotoxin 3-Hydroxykynurenine in the Frontal Cortex of HIV-1-Positive Patients. J. Neurochem..

[138] Okuda S., Nishiyama N., Saito H., Katsuki H. (1996). Hydrogen peroxide-mediated neuronal cell death induced by an endogenous neurotoxin, 3-hydroxykynurenine. Proc. Natl. Acad. Sci. USA.

[139] Colín-González A.L., Maya-López M., Pedraza-Chaverrí J., Ali S.F., Chavarría A., Santamaría A. (2014). The Janus faces of 3-hydroxykynurenine: Dual redox modulatory activity and lack of neurotoxicity in the rat striatum. Brain Res..

[140] Nakagami Y., Saito H., Katsuki H. (1996). 3-Hydroxykynurenine toxicity on the rat striatum in vivo. Jpn. J. Pharmacol..

[141] Guidetti P., Schwarcz R. (1999). 3-Hydroxykynurenine potentiates quinolinate but not NMDA toxicity in the rat striatum. Eur. J. Neurosci..

[142] Darlington L.G., Forrest C.M., Mackay G.M., Smith R.A., Smith A.J., Stoy N., Stone T.W. (2010). On the biological importance of the 3-hydroxyanthranilic acid: Anthranilic acid ratio. Int. J. Tryptophan Res..

[143] Ramírez-Ortega D., Ramiro-Salazar A., González-Esquivel D., Ríos C., Pineda B., Pérez De La Cruz V. (2017). 3-Hydroxykynurenine and 3-Hydroxyanthranilic Acid Enhance the Toxicity Induced By Copper in Rat Astrocyte Culture. Oxid. Med. Cell. Longev..

[144] Gadupudi G.S., Chung K.T. (2011). Comparative genotoxicity of 3-hydroxyanthranilic acid and anthranilic acid in the presence of a metal cofactor Cu (II) in vitro. Mutat. Res. Genet. Toxicol. Environ. Mutagen..

[145] Krause D., Suh H.S., Tarassishin L., Cui Q.L., Durafourt B.A., Choi N., Bauman A., Cosenza-Nashat M., Antel J.P., Zhao M.L. (2011). The tryptophan metabolite 3-hydroxyanthranilic acid plays anti-inflammatory and neuroprotective roles during inflammation: Role of hemeoxygenase-1. Am. J. Pathol..

[146] Reyes-Ocampo J., Ramírez-Ortega D., Vázquez Cervantes G.I., Pineda B., Montes de Oca Balderas P., González-Esquivel D., Sánchez-Chapul L., Lugo-Huitrón R., Silva-Adaya D., Ríos C. (2015). Mitochondrial dysfunction related to cell damage induced by 3-hydroxykynurenine and 3-hydroxyanthranilic acid: Non-dependent-effect of early reactive oxygen species production. Neurotoxicology.

[147] Berg M., Polyzos K.A., Agardh H., Baumgartner R., Forteza M.J., Kareinen I., Gisterå A., Bottcher G., Hurt-Camejo E., Hansson G.K. (2019). 3-Hydroxyanthralinic acid metabolism controls the hepatic SREBP/lipoprotein axis, inhibits inflammasome activation in macrophages, and decreases atherosclerosis in Ldlr^−/−^ mice. Cardiovasc. Res..

[148] Stone T.W., Perkins M.N. (1981). Quinolinic acid: A potent endogenous excitant at amino acid receptors in CNS. Eur. J. Pharmacol..

[149] Guillemin G.J., Brew B.J., Noonan C.E., Takikawa O., Cullen K.M. (2005). Indoleamine 2,3 dioxygenase and quinolinic acid Immunoreactivity in Alzheimer’s disease hippocampus. Neuropathol. Appl. Neurobiol..

[150] Beal M.F., Ferrante R.J., Swartz K.J., Kowall N.W. (1991). Chronic quinolinic acid lesions in rats closely resemble Huntington’s disease. J. Neurosci..

[151] Heyes M.P. (2001). Elevated cerebrospinal fluid quinolinic acid levels are associated with region-specific cerebral volume loss in HIV infection. Brain.

[152] Yan E.B., Frugier T., Lim C.K., Heng B., Sundaram G., Tan M., Rosenfeld J.V., Walker D.W., Guillemin G.J., Morganti-Kossmann M.C. (2015). Activation of the kynurenine pathway and increased production of the excitotoxin quinolinic acid following traumatic brain injury in humans. J. Neuroinflammation.

[153] Moroni F., Cozzi A., Carpendo R., Cipriani G., Veneroni O., Izzo E. (2005). Kynurenine 3-mono-oxygenase inhibitors reduce glutamate concentration in the extracellular spaces of the basal ganglia but not in those of the cortex or hippocampus. Neuropharmacology.

[154] Brundin L., Sellgren C.M., Lim C.K., Grit J., Pålsson E., Landén M., Samuelsson M., Lundgren K., Brundin P., Fuchs D. (2016). An enzyme in the kynurenine pathway that governs vulnerability to suicidal behavior by regulating excitotoxicity and neuroinflammation. Transl. Psychiatry.

[155] Rahman A., Rao M.S., Khan K.M. (2018). Intraventricular infusion of quinolinic acid impairs spatial learning and memory in young rats: A novel mechanism of lead-induced neurotoxicity. J. Neuroinflammation.

[156] Latif-hernandez A., Shah D., Ahmed T., Lo A.C., Callaerts- Z. (2016). Quinolinic acid injection in mouse medial prefrontal cortex affects reversal learning abilities, cortical connectivity and hippocampal synaptic plasticity. Nat. Publ. Gr..

[157] Savitz J., Drevets W.C., Wurfel B.E., Ford B.N., Bellgowan P.S.F., Victor T.A., Bodurka J., Teague T.K., Dantzer R. (2015). Reduction of kynurenic acid to quinolinic acid ratio in both the depressed and remitted phases of major depressive disorder. Brain. Behav. Immun..

[158] Prado de Carvalho L., Bochet P., Rossier J. (1996). The endogeneous agonist quinolinic acid and the non endogenous homoquinolinic acid discriminate between nmdar2 receptor subunits. Neurochem. Int..

[159] Pierozan P., Ferreira F., de Lima B.O., Pessoa-Pureur R. (2015). Quinolinic acid induces disrupts cytoskeletal homeostasis in striatal neurons. Protective role of astrocyte-neuron interaction. J. Neurosci. Res..

[160] Guillemin G.J., Croitoru-Lamoury J., Dormont D., Armati P.J., Brew B.J. (2003). Quinolinic acid upregulates chemokine production and chemokine receptor expression in astrocytes. Glia.

[161] Speciale C., Hares K., Schwarcz R., Brookes N. (1989). High-affinity uptake of L-kynurenine by a Na+-independent transporter of neutral amino acids in astrocytes. J. Neurosci..

[162] Ramos-Chávez L.A., Lugo Huitrón R., González Esquivel D., Pineda B., Ríos C., Silva-Adaya D., Sánchez-Chapul L., Roldán-Roldán G., Pérez de la Cruz V. (2018). Relevance of alternative routes of kynurenic acid production in the brain. Oxid. Med. Cell. Longev..

[163] Wang J., Simonavicius N., Wu X., Swaminath G., Reagan J., Tian H., Ling L. (2006). Kynurenic acid as a ligand for orphan G protein-coupled receptor GPR35. J. Biol. Chem..

[164] Beggiato S., Tanganelli S., Fuxe K., Antonelli T., Schwarcz R., Ferraro L. (2014). Endogenous kynurenic acid regulates extracellular GABA levels in the rat prefrontal cortex. Neuropharmacology.

[165] Zmarowski A., Wu H.Q., Brooks J.M., Potter M.C., Pellicciari R., Schwarcz R., Bruno J.P. (2009). Astrocyte-derived kynurenic acid modulates basal and evoked cortical acetylcholine release. Eur. J. Neurosci..

[166] Flores-Barrera E., Thomases D.R., Cass D.K., Bhandari A., Schwarcz R., Bruno J.P., Tseng K.Y. (2017). Preferential disruption of prefrontal GABAergic function by nanomolar concentrations of the α7nACh negative modulator kynurenic acid. J. Neurosci..

[167] Carpenedo R., Pittaluga A., Cozzi A., Attucci S., Galli A., Raiteri M., Moroni F. (2001). Presynaptic kynurenate-sensitive receptors inhibit glutamate release. Eur. J. Neurosci..

[168] Konradsson-Geuken Å., Wu H.Q., Gash C.R., Alexander K.S., Campbell A., Sozeri Y., Pellicciari R., Schwarcz R., Bruno J.P. (2010). Cortical kynurenic acid bi-directionally modulates prefrontal glutamate levels as assessed by microdialysis and rapid electrochemistry. Neuroscience.

[169] Sathyasaikumar K.V., Stachowski E.K., Wonodi I., Roberts R.C., Rassoulpour A., McMahon R.P., Schwarcz R. (2011). Impaired kynurenine pathway metabolism in the prefrontal cortex of individuals with schizophrenia. Schizophr. Bull..

[170] Alexander K.S., Wu H.Q., Schwarcz R., Bruno J.P. (2012). Acute elevations of brain kynurenic acid impair cognitive flexibility: Normalization by the alpha7 positive modulator galantamine. Psychopharmacology.

[171] Banerjee J., Alkondon M., Pereira E.F.R., Albuquerque E.X. (2012). Regulation of GABAergic inputs to CA1 pyramidal neurons by nicotinic receptors and kynurenic acid. J. Pharmacol. Exp. Ther..

[172] Bortz D.M., Wu H.Q., Schwarcz R., Bruno J.P. (2017). Oral administration of a specific kynurenic acid synthesis (KAT II) inhibitor attenuates evoked glutamate release in rat prefrontal cortex. Neuropharmacology.

[173] Koshy Cherian A., Gritton H., Johnson D.E., Young D., Kozak R., Sarter M. (2014). A systemically-available kynurenine aminotransferase II (KAT II) inhibitor restores nicotine-evoked glutamatergic activity in the cortex of rats. Neuropharmacology.

[174] Potter M.C., Elmer G.I., Bergeron R., Albuquerque E.X., Guidetti P., Wu H.Q., Schwarcz R. (2010). Reduction of endogenous kynurenic acid formation enhances extracellular glutamate, hippocampal plasticity, and cognitive behavior. Neuropsychopharmacology.

[175] Erhardt S., Schwieler L., Emanuelsson C., Geyer M. (2004). Endogenous kynurenic acid disrupts prepulse inhibition. Biol. Psychiatry.

[176] Linderholm K.R., Skogh E., Olsson S.K., Dahl M.L., Holtze M., Engberg G., Samuelsson M., Erhardt S. (2012). Increased levels of kynurenine and kynurenic acid in the CSF of patients with schizophrenia. Schizophr. Bull..

[177] Olsson S.K., Samuelsson M., Saetre P., Lindström L., Jönsson E.G., Nordin C., Engberg G., Erhardt S., Landén M. (2010). Elevated levels of kynurenic acid in the cerebrospinal fluid of patients with bipolar disorder. J. Psychiatry Neurosci..

[178] Sellgren C.M., Gracias J., Jungholm O., Perlis R.H., Engberg G., Schwieler L., Landen M., Erhardt S. (2019). Peripheral and central levels of kynurenic acid in bipolar disorder subjects and healthy controls. Transl. Psychiatry.

[179] Berlinguer-Palmini R., Masi A., Narducci R., Cavone L., Maratea D., Cozzi A., Sili M., Moroni F., Mannaioni G. (2013). GPR35 activation reduces Ca^2+^ transients and contributes to the kynurenic acid-dependent reduction of synaptic activity at CA3-CA1 synapses. PLoS ONE.

[180] Pocivavsek A., Wu H.Q., Potter M.C., Elmer G.I., Pellicciari R., Schwarcz R. (2011). Fluctuations in endogenous kynurenic acid control hippocampal glutamate and memory. Neuropsychopharmacology.

[181] Vohra M., Lemieux G.A., Lin L., Ashrafi K. (2018). Kynurenic acid accumulation underlies learning and memory impairment associated with aging. Genes Dev..

[182] Peyton L., Oliveros A., Tufvesson-Alm M., Schwieler L., Starski P., Engberg G., Erhardt S., Choi D.S. (2019). Lipopolysaccharide Increases Cortical Kynurenic Acid and Deficits in Reference Memory in Mice. Int. J. Tryptophan Res..

[183] Wennström M., Nielsen H.M., Orhan F., Londos E., Minthon L., Erhardt S. (2014). Kynurenic acid levels in cerebrospinal fluid from patients with alzheimer’s disease or dementia with lewy bodies. Int. J. Tryptophan Res..

[184] González-Sánchez M., Jiménez J., Narváez A., Antequera D., Llamas-Velasco S., Martín A.H.S., Arjona J.A.M., de Munain A.L., Bisa A.L., Marco M.P. (2020). Kynurenic acid levels are increased in the CSF of Alzheimer’s disease patients. Biomolecules.

[185] Chatterjee P., Zetterberg H., Goozee K., Lim C.K., Jacobs K.R., Ashton N.J., Hye A., Pedrini S., Sohrabi H.R., Shah T. (2019). Plasma neurofilament light chain and amyloid-β are associated with the kynurenine pathway metabolites in preclinical Alzheimer’s disease. J. Neuroinflammation.

[186] Giil L.M., Midttun Ø., Refsum H., Ulvik A., Advani R., Smith A.D., Ueland P.M. (2017). Kynurenine Pathway Metabolites in Alzheimer’s Disease. J. Alzheimer’s Dis..

[187] Pierozan P., Biasibetti-brendler H., Schmitz F., Ferreira F., Pessoa-pureur R., Wyse A.T.S. (2017). Kynurenic Acid Prevents Cytoskeletal Disorganization Induced by Quinolinic Acid in Mixed Cultures of Rat Striatum. Mol. Neurobiol..

[188] Gobaille S., Kemmel V., Brumaru D., Dugave C., Aunis D., Maitre M. (2008). Xanthurenic acid distribution, transport, accumulation and release in the rat brain. J. Neurochem..

[189] Uwai Y., Honjo E. (2013). Transport of xanthurenic acid by rat/human organic anion transporters OAT1 and OAT3. Biosci. Biotechnol. Biochem..

[190] Copeland C.S., Neale S.A., Salt T.E. (2013). Actions of Xanthurenic Acid, a putative endogenous Group II metabotropic glutamate receptor agonist, on sensory transmission in the thalamus. Neuropharmacology.

[191] Hikichi H., Hiyoshi T., Marumo T., Tomishima Y., Kaku A., Iida I., Urabe H., Tamita T., Yasuhara A., Karasawa J.I. (2015). Antipsychotic profiles of TASP0443294, a novel and orally active positive allosteric modulator of metabotropic glutamate 2 receptor. J. Pharmacol. Sci..

[192] Moghaddam B., Adams B.W. (1998). Reversal of phencyclidine effects by a group II metabotropic glutamate receptor agonist in rats. Science.

[193] Fazio F., Carrizzo A., Lionetto L., Damato A., Capocci L., Ambrosio M., Battaglia G., Bruno V., Madonna M., Simmaco M. (2017). Vasorelaxing action of the kynurenine metabolite, xanthurenic acid: The missing link in endotoxin-induced hypotension?. Front. Pharmacol..

[194] Nagamura Y., Uesugi K., Naito J., Ishiguro I. (1999). Cinnabarinic acid was formed in damaged mitochondria and its effect on mitochondrial respiration. Adv. Exp. Med. Biol..

[195] Joshi A.D., Hossain E., Elferink C.J. (2017). Epigenetic regulation by agonist-specific aryl hydrocarbon receptor recruitment of metastasis-associated protein 2 selectively induces stanniocalcin 2 expression. Mol. Pharmacol..

[196] Braidy N., Guillemin G.J., Mansour H., Chan-Ling T., Grant R. (2011). Changes in kynurenine pathway metabolism in the brain, liver and kidney of aged female Wistar rats. FEBS J..

[197] Grant R.S., Coggan S.E., Smythe G.A. (2009). The physiological action of picolinic acid in the human brain. Int. J. Tryptophan Res..

[198] Bosco M.C., Rapisarda A., Massazza S., Melillo G., Young H., Varesio L. (2000). The Tryptophan Catabolite Picolinic Acid Selectively Induces the Chemokines Macrophage Inflammatory Protein-1α and -1β in Macrophages. J. Immunol..

[199] Prodinger J., Loacker L.J., Schmidt R.L.J., Ratzinger F., Greiner G., Witzeneder N., Hoermann G., Jutz S., Pickl W.F., Steinberger P. (2016). The tryptophan metabolite picolinic acid suppresses proliferation and metabolic activity of CD^4+^ T cells and inhibits c-Myc activation. J. Leukoc. Biol..

[200] Beskid M., Jachimowicz J., Taraszewska A., Kukulska D. (1995). Histological and ultrastructural changes in the rat brain following systemic administration of picolinic acid. Exp. Toxicol. Pathol..

[201] Vrooman L., Jhamandas K., Boegman R.J., Beninger R.J. (1993). Picolinic acid modulates kainic acid-evoked glutamate release from the striatum in vitro. Brain Res..

[202] Lapin I.P. (1978). Stimulant and convulsive effects of kynurenines injected into brain ventricles in mice. J. Neural Transm..

[203] Ryan K.M., Allers K.A., McLoughlin D.M., Harkin A. (2020). Tryptophan metabolite concentrations in depressed patients before and after electroconvulsive therapy. Brain. Behav. Immun..

[204] Dantzer R., O’Connor J.C., Freund G.G., Johnson R.W., Kelley K.W. (2008). From inflammation to sickness and depression: When the immune system subjugates the brain. Nat. Rev. Neurosci..

[205] Menard C., Pfau M.L., Hodes G.E., Kana V., Wang V.X., Bouchard S., Takahashi A., Flanigan M.E., Aleyasin H., Leclair K.B. (2017). Social stress induces neurovascular pathology promoting depression. Nat. Neurosci..

[206] Chen W.W., Zhang X., Huang W.J. (2016). Role of neuroinflammation in neurodegenerative diseases (Review). Mol. Med. Rep..

[207] Ransohoff R.M. (2016). How neuroinflammation contributes to neurodegeneration. Science.

[208] Morales I., Farías G.A., Cortes N., Maccioni R.B. (2016). Neuroinflammation and Neurodegeneration. Update on Dementia.

[209] Boche D., Perry V.H., Nicoll J.A.R. (2013). Review: Activation patterns of microglia and their identification in the human brain. Neuropathol. Appl. Neurobiol..

[210] Butovsky O., Weiner H.L. (2018). Microglial signatures and their role in health and disease. Nat. Rev. Neurosci..

[211] Konishi H., Kiyama H. (2018). Microglial TREM2/DAP12 signaling: A double-edged sword in neural diseases. Front. Cell. Neurosci..

[212] Gratuze M., Leyns C.E.G., Holtzman D.M. (2018). New insights into the role of TREM2 in Alzheimer’s disease. Mol. Neurodegener..

[213] Hu Y., Fryatt G.L., Ghorbani M., Obst J., Menassa D.A., Martin-Estebane M., Muntslag T.A.O., Olmos-Alonso A., Guerrero-Carrasco M., Thomas D. (2021). Replicative senescence dictates the emergence of disease-associated microglia and contributes to Aβ pathology. Cell Rep..

[214] Moraga-Amaro R., Jerez-Baraona J.M., Simon F., Stehberg J. (2014). Role of astrocytes in memory and psychiatric disorders. J. Physiol. Paris.

[215] Pekny M., Pekna M. (2016). Reactive gliosis in the pathogenesis of CNS diseases. Biochim. Biophys. Acta Mol. Basis Dis..

[216] Phatnani H., Maniatis T. (2015). Astrocytes in neurodegenerative disease. Cold Spring Harb. Perspect. Biol..

[217] Jana M., Palencia C.A., Pahan K. (2008). Fibrillar Amyloid-β Peptides Activate Microglia via TLR2: Implications for Alzheimer’s Disease. J. Immunol..

[218] Liu S., Liu Y., Hao W., Wolf L., Kiliaan A.J., Penke B., Rübe C.E., Walter J., Heneka M.T., Hartmann T. (2012). TLR2 Is a Primary Receptor for Alzheimer’s Amyloid β Peptide To Trigger Neuroinflammatory Activation. J. Immunol..

[219] Souza L.C., Jesse C.R., Antunes M.S., Ruff J.R., de Oliveira Espinosa D., Gomes N.S., Donato F., Giacomeli R., Boeira S.P. (2016). Indoleamine-2,3-dioxygenase mediates neurobehavioral alterations induced by an intracerebroventricular injection of amyloid-β1-42 peptide in mice. Brain. Behav. Immun..

[220] Mazarei G., Budac D.P., Lu G., Lee H., Möller T., Leavitt B.R. (2013). The absence of indoleamine 2,3-dioxygenase expression protects against NMDA receptor-mediated excitotoxicity in mouse brain. Exp. Neurol..

[221] Hochstrasser T., Ullrich C., Sperner-Unterweger B., Humpel C. (2011). Inflammatory stimuli reduce survival of serotonergic neurons and induce neuronal expression of indoleamine 2,3-dioxygenase in rat dorsal raphe nucleus organotypic brain slices. Neuroscience.

[222] O’Farrell K., Fagan E., Connor T.J., Harkin A. (2017). Inhibition of the kynurenine pathway protects against reactive microglial-associated reductions in the complexity of primary cortical neurons. Eur. J. Pharmacol..

[223] Feng W., Wang Y., Qi Z., Xuan L., Han R., Zhu Y. (2017). Microglia activation contributes to quinolinic acid-induced neuronal excitotoxicity through TNF-α. Apoptosis.

[224] Mangas A., Yajeya J., González N., Ruiz I., Pernía M., Geffard M., Coveñas R. (2017). Gemst: A taylor-made combination that reverts neuroanatomical changes in stroke. Eur. J. Histochem..

[225] Xie W., Cai L., Yu Y., Gao L., Xiao L., He Q., Ren Z., Liu Y. (2014). Activation of brain indoleamine 2,3-dioxygenase contributes to epilepsy-associated depressive-like behavior in rats with chronic temporal lobe epilepsy. J. Neuroinflammation.

[226] Dobos N., de Vries E.F.J., Kema I.P., Patas K., Prins M., Nijholt I.M., Dierckx R.A., Korf J., den Boer J.A., Luiten P.G.M. (2012). The Role of Indoleamine 2,3-Dioxygenase in a Mouse Model of Neuroinflammation-Induced Depression. J. Alzheimer’s Dis..

[227] Yu D., Tao B.-B., Yang Y.-Y., Du L.-S., Yang S.-S., He X.-J., Zhu Y.-W., Yan J.-K., Yang Q. (2015). The IDO Inhibitor Coptisine Ameliorates Cognitive Impairment in a Mouse Model of Alzheimer’s Disease. J. Alzheimer’s Dis..

[228] Grégoire L., Rassoulpour A., Guidetti P., Samadi P., Bédard P.J., Izzo E., Schwarcz R., Di Paolo T. (2008). Prolonged kynurenine 3-hydroxylase inhibition reduces development of levodopa-induced dyskinesias in parkinsonian monkeys. Behav. Brain Res..

[229] Richter A., Hamann M. (2003). The kynurenine 3-hydroxylase inhibitor Ro 61-8048 improves dystonia in a genetic model of paroxysmal dyskinesia. Eur. J. Pharmacol..

[230] Zwilling D., Huang S.-Y., Sathyasaikumar K.V., Notarangelo F.M., Guidetti P., Wu H.-Q., Lee J., Truong J., Andrews-Zwilling Y., Hsieh E.W. (2011). Kynurenine 3-Monooxygenase Inhibition in Blood Ameliorates Neurodegeneration. Cell.

[231] Guidetti P., Wu H.Q., Schwarcz R. (2000). In situ produced 7-chlorokynurenate provides protection against quinolinate- and malonate-induced neurotoxicity in the rat striatum. Exp. Neurol..

[232] Zádori D., Klivényi P., Szalárdy L., Fülöp F., Toldi J., Vécsei L. (2012). Mitochondrial disturbances, excitotoxicity, neuroinflammation and kynurenines: Novel therapeutic strategies for neurodegenerative disorders. J. Neurol. Sci..

[233] Gellért L., Fuzik J., Göblös A., Sárközi K., Marosi M., Kis Z., Farkas T., Szatmári I., Fülöp F., Vécsei L. (2011). Neuroprotection with a new kynurenic acid analog in the four-vessel occlusion model of ischemia. Eur. J. Pharmacol..

[234] Demeter I., Nagy K., Gellért L., Vécsei L., Fülöp F., Toldi J. (2012). A novel kynurenic acid analog (SZR104) inhibits pentylenetetrazole-induced epileptiform seizures. An electrophysiological study: Special issue related to Kynurenine. J. Neural Transm..

[235] Zakhary G., Sherchan P., Li Q., Tang J., Zhang J.H. (2020). Modification of kynurenine pathway via inhibition of kynurenine hydroxylase attenuates surgical brain injury complications in a male rat model. J. Neurosci. Res..

[236] Beaumont V., Mrzljak L., Dijkman U., Freije R., Heins M., Rassoulpour A., Tombaugh G., Gelman S., Bradaia A., Steidl E. (2016). The novel KMO inhibitor CHDI-340246 leads to a restoration of electrophysiological alterations in mouse models of Huntington’s disease. Exp. Neurol..

[237] Wu H.Q., Guidetti P., Goodman J.H., Varasi M., Ceresoli-Borroni G., Speciale C., Scharfman H.E., Schwarcz R. (2000). Kynurenergic manipulations influence excitatory synaptic function and excitotoxic vulnerability in the rat hippocampus in vivo. Neuroscience.

[238] Walker A.K., Wing E.E., Banks W.A., Dantzer R. (2019). Leucine competes with kynurenine for blood-to-brain transport and prevents lipopolysaccharide-induced depression-like behavior in mice. Mol. Psychiatry.

[239] Schwieler L., Samuelsson M., Frye M.A., Bhat M., Schuppe-Koistinen I., Jungholm O., Johansson A.G., Landén M., Sellgren C.M., Erhardt S. (2016). Electroconvulsive therapy suppresses the neurotoxic branch of the kynurenine pathway in treatment-resistant depressed patients. J. Neuroinflammation.

[240] Guloksuz S., Arts B., Walter S., Drukker M., Rodriguez L., Myint A.M., Schwarz M.J., Ponds R., van Os J., Kenis G. (2015). The impact of electroconvulsive therapy on the tryptophan-kynurenine metabolic pathway. Brain. Behav. Immun..

[241] Zhou Y., Zheng W., Liu W., Wang C., Zhan Y., Li H., Chen L., Li M., Ning Y. (2018). Antidepressant effect of repeated ketamine administration on kynurenine pathway metabolites in patients with unipolar and bipolar depression. Brain. Behav. Immun..

[242] Allen A.P., Naughton M., Dowling J., Walsh A., O’Shea R., Shorten G., Scott L., McLoughlin D.M., Cryan J.F., Clarke G. (2018). Kynurenine pathway metabolism and the neurobiology of treatment-resistant depression: Comparison of multiple ketamine infusions and electroconvulsive therapy. J. Psychiatr. Res..

[243] Wu H.Q., Lee S.C., Scharfman H.E., Schwarcz R. (2002). L-4-chlorokynurenine attenuates kainate-induced seizures and lesions in the rat. Exp. Neurol..

[244] Zanos P., Piantadosi S.C., Wu H.Q., Pribut H.J., Dell M.J., Can A., Snodgrass H.R., Zarate C.A., Schwarcz R., Gould T.D. (2015). The prodrug 4-chlorokynurenine causes ketamine-like antidepressant effects, but not side effects, by NMDA/glycineB-site inhibitionS. J. Pharmacol. Exp. Ther..

[245] Wallace M., White A., Grako K.A., Lane R., Cato A. (2017). (Jo); Snodgrass, H.R. Randomized, double-blind, placebo-controlled, dose-escalation study: Investigation of the safety, pharmacokinetics, and antihyperalgesic activity of L-4-chlorokynurenine in healthy volunteers. Scand. J. Pain.

[246] Halaris A., Myint A.M., Savant V., Meresh E., Lim E., Guillemin G., Hoppensteadt D., Fareed J., Sinacore J. (2015). Does escitalopram reduce neurotoxicity in major depression?. J. Psychiatr. Res..

[247] Castillo M.F.R., Murata S., Schwarz M., Schütze G., Moll N., Martin B., Burger B., Weidinger E., Mueller N., Halaris A. (2019). Celecoxib augmentation of escitalopram in treatment-resistant bipolar depression and the effects on Quinolinic Acid. Neurol. Psychiatry Brain Res..

[248] Kocki T., Urbańska E.M., Kocki J., Kloc R., Kocka K., Olajossy M., Owe-Larsson B. (2018). Prolonged therapy with antidepressants increases hippocampal level of kynurenic acid and expression of Kat1 and Kat2 genes. Pharmacol. Reports.

[249] Réus G.Z., Becker I.R.T., Scaini G., Petronilho F., Oses J.P., Kaddurah-Daouk R., Ceretta L.B., Zugno A.I., Dal-Pizzol F., Quevedo J. (2018). The inhibition of the kynurenine pathway prevents behavioral disturbances and oxidative stress in the brain of adult rats subjected to an animal model of schizophrenia. Prog. Neuro-Psychopharmacol. Biol. Psychiatry.

[250] Gibney S.M., Fagan E.M., Waldron A.M., O’Byrne J., Connor T.J., Harkin A. (2014). Inhibition of stress-induced hepatic tryptophan 2,3-dioxygenase exhibits antidepressant activity in an animal model of depressive behaviour. Int. J. Neuropsychopharmacol..

[251] Guillemin G.J., Smythe G., Takikawa O., Brew B.J. (2005). Expression of indoleamine 2,3-dioxygenase and production of quinolinic acid by human microglia, astrocytes, and neurons. Glia.

[252] Fuchs D., Reibnegger G., Werner E.R., Wachter H., Forsman A., Larsson M., Hagberg L., Norkrans G. (1990). Immune Activation and Decreased Tryptophan in Patients with HIV-1 Infection. J. Interferon Res..

[253] Bonda D.J., Mailankot M., Stone J.G., Garrett M.R., Staniszewska M., Castellani R.J., Siedlak S.L., Zhu X., Lee H.G., Perry G. (2010). Indoleamine 2,3-dioxygenase and 3-hydroxykynurenine modifications are found in the neuropathology of Alzheimer’s disease. Redox Rep..

[254] Breda C., Sathyasaikumar K.V., Idrissi S.S., Notarangelo F.M., Estranero J.G., Moore G.G.L., Green E.W., Kyriacou C.P., Schwarcz R., Giorgini F. (2016). Tryptophan-2,3-dioxygenase (TDO) inhibition ameliorates neurodegeneration by modulation of kynurenine pathway metabolites. Proc. Natl. Acad. Sci. USA.

[255] Cozzi A., Carpenedo R., Moroni F. (1999). Kynurenine hydroxylase inhibitors reduce ischemic brain damage: Studies with (m-nitrobenzoyl)-alanine (mNBA) and 3,4-dimethoxy-[-N-4-(nitrophenyl)thiazol-2YL]-benzenesulfonamide (Ro 61-8048) in models of focal or global brain ischemia. J. Cereb. Blood Flow Metab..

[256] Zádori D., Nyiri G., Szonyi A., Szatmári I., Fülöp F., Toldi J., Freund T.F., Vécsei L., Klivényi P. (2011). Neuroprotective effects of a novel kynurenic acid analogue in a transgenic mouse model of Huntington’s disease. J. Neural Transm..

[257] Capuron L., Ravaud A., Dantzer R. (2000). Early depressive symptoms in cancer patients receiving interleukin 2 and/or interferon alfa-2b therapy. J. Clin. Oncol..

[258] Breit S., Kupferberg A., Rogler G., Hasler G. (2018). Vagus nerve as modulator of the brain-gut axis in psychiatric and inflammatory disorders. Front. Psychiatry.

[259] Miller A.H., Raison C.L. (2016). The role of inflammation in depression: From evolutionary imperative to modern treatment target. Nat. Rev. Immunol..

[260] Salazar A., Gonzalez-Rivera B.L., Redus L., Parrott J.M., O’Connor J.C. (2012). Indoleamine 2,3-dioxygenase mediates anhedonia and anxiety-like behaviors caused by peripheral lipopolysaccharide immune challenge. Horm. Behav..

[261] Sun Y., Drevets W., Turecki G., Li Q.S. (2020). The relationship between plasma serotonin and kynurenine pathway metabolite levels and the treatment response to escitalopram and desvenlafaxine. Brain. Behav. Immun..

[262] Eskelund A., Li Y., Budac D.P., Müller H.K., Gulinello M., Sanchez C., Wegener G. (2017). Drugs with antidepressant properties affect tryptophan metabolites differently in rodent models with depression-like behavior. J. Neurochem..

[263] Liu Y.N., Peng Y.L., Liu L., Wu T.Y., Zhang Y., Lian Y.J., Yang Y.Y., Kelley K.W., Jiang C.L., Wang Y.X. (2015). TNFα mediates stress-induced depression by upregulating indoleamine 2,3-dioxygenase in a mouse model of unpredictable chronic mild stress. Eur. Cytokine Netw..

[264] Martín-Hernández D., Tendilla-Beltrán H., Madrigal J.L.M., García-Bueno B., Leza J.C., Caso J.R. (2019). Chronic Mild Stress Alters Kynurenine Pathways Changing the Glutamate Neurotransmission in Frontal Cortex of Rats. Mol. Neurobiol..

[265] Steiner J., Walter M., Gos T., Guillemin G.J., Bernstein H.G., Sarnyai Z., Mawrin C., Brisch R., Bielau H., zu Schwabedissen L.M. (2013). Correction to Severe depression is associated with increased microglial quinolinic acid in subregions of the anterior cingulate gyrus: Evidence for an immune-modulated glutamatergic neurotransmission?. J. Neuroinflamm..

[266] Young K.D., Drevets W.C., Dantzer R., Teague T.K., Bodurka J., Savitz J. (2016). Kynurenine pathway metabolites are associated with hippocampal activity during autobiographical memory recall in patients with depression. Brain. Behav. Immun..

[267] Wilkinson S.T., Ballard E.D., Bloch M.H., Mathew S.J., Murrough J.W., Feder A., Sos P., Wang G., Zarate C.A., Sanacora G. (2018). The effect of a single dose of intravenous ketamine on suicidal ideation: A systematic review and individual participant data meta-analysis. Am. J. Psychiatry.

[268] Haroon E., Welle J.R., Woolwine B.J., Goldsmith D.R., Baer W., Patel T., Felger J.C., Miller A.H. (2020). Associations among peripheral and central kynurenine pathway metabolites and inflammation in depression. Neuropsychopharmacology.

[269] Kim H., Chen L., Lim G., Sung B., Wang S., Mccabe M.F. (2012). Contributes To the Comorbidity of Pain and Depression. J. Clin. Invest..

[270] Hemmati S., Sadeghi M.A., Mohammad Jafari R., Yousefi-Manesh H., Dehpour A.R. (2019). The antidepressant effects of GM-CSF are mediated by the reduction of TLR4/NF-KB-induced IDO expression. J. Neuroinflammation.

[271] Seiler C., Avila C., Khana R., Springer E., James C., Armstrong D., Marshall J., Collins S.M., Pinto-Sanchez M.I., Bercik P. (2018). A67 Rapid Reduction In Anxiety Scores In Ibd Patients After Infliximab Infusion Is Associated With Changes In Kynurenine/Tryptophan Metabolism. J. Can. Assoc. Gastroenterol..

[272] Wonodi I., Stine O.C., Sathyasaikumar K.V., Roberts R.C., Mitchell B.D., Hong L.E., Kajii Y., Thaker G.K., Schwarcz R. (2011). Downregulated kynurenine 3-monooxygenase gene expression and enzyme activity in schizophrenia and genetic association with schizophrenia endophenotypes. Arch. Gen. Psychiatry.

[273] Wonodi I., McMahon R.P., Krishna N., Mitchell B.D., Liu J., Glassman M., Elliot Hong L., Gold J.M. (2014). Influence of kynurenine 3-monooxygenase (KMO) gene polymorphism on cognitive function in schizophrenia. Schizophr. Res..

[274] Lavebratt C., Olsson S., Backlund L., Frisén L., Sellgren C., Priebe L., Nikamo P., Träskman-Bendz L., Cichon S., Vawter M.P. (2014). The KMO allele encoding Arg 452 is associated with psychotic features in bipolar disorder type 1, and with increased CSF KYNA level and reduced KMO expression. Mol. Psychiatry.

[275] Aoyama N., Takahashi N., Saito S., Maeno N., Ishihara R., Ji X., Miura H., Ikeda M., Suzuki T., Kitajima T. (2006). Association study between kynurenine 3-monooxygenase gene and schizophrenia in the Japanese population. Genes Brain Behav..

[276] Lezheiko T.V., Golimbet V.E., Andryushchenko A.V., Melik-Pashayan A.E., Mironova E.V. (2018). Studies of the Association between the Kynurenine-3-Monooxygenase Gene and Depression. Neurosci. Behav. Physiol..

[277] Douet V., Tanizaki N., Franke A., Li X., Chang L. (2016). Polymorphism of Kynurenine Pathway-Related Genes, Kynurenic Acid, and Psychopathological Symptoms in HIV. J. Neuroimmune Pharmacol..

[278] Claes S., Myint A.M., Domschke K., Del-Favero J., Entrich K., Engelborghs S., De Deyn P., Mueller N., Baune B., Rothermundt M. (2011). The kynurenine pathway in major depression: Haplotype analysis of three related functional candidate genes. Psychiatry Res..

[279] Cutler J.A., Rush A.J., McMahon F.J., Laje G. (2012). Common genetic variation in the indoleamine-2,3-dioxygenase genes and antidepressant treatment outcome in major depressive disorder. J. Psychopharmacol..

[280] Wang S.Y., Duan K.M., Tan X.F., Yin J.Y., Mao X.Y., Zheng W., Wang C.Y., Yang M., Peng C., Zhou H.H. (2017). Genetic variants of the kynurenine-3-monooxygenase and postpartum depressive symptoms after cesarean section in Chinese women. J. Affect. Disord..

[281] Duan K.M., Wang S.Y., Yin J.Y., Li X., Ma J.H., Huang Z.D., Zhou Y.Y., Yu H.Y., Yang M., Zhou H.H. (2019). The IDO genetic polymorphisms and postpartum depressive symptoms: An association study in Chinese parturients who underwent cesarean section. Arch. Womens. Ment. Health.

[282] Quan C., Wang S., Duan K., Ma J., Yu H., Yang M., Hu N., Long G., Zeng G., Huang Z. (2020). The role of kynurenine pathway and kynurenic aminotransferase alleles in postpartum depression following cesarean section in Chinese women. Brain Behav..

[283] Martí-Massó J.F., Bergareche A., Makarov V., Ruiz-Martinez J., Gorostidi A., De Munain A.L., Poza J.J., Striano P., Buxbaum J.D., Paisán-Ruiz C. (2013). The ACMSD gene, involved in tryptophan metabolism, is mutated in a family with cortical myoclonus, epilepsy, and parkinsonism. J. Mol. Med..

[284] Vilas D., Fernández-Santiago R., Sanchez E., Azcona L.J., Santos-Montes M., Casquero P., Argandoña L., Tolosa E., Paisán-Ruiz C. (2017). A Novel p.Glu298Lys Mutation in the ACMSD Gene in Sporadic Parkinson’s Disease. J. Parkinsons. Dis..

[285] Nalls M.A., Plagnol V., Hernandez D.G., Sharma M., Sheerin U.M., Saad M., Simón-Sánchez J., Schulte C., Lesage S., Sveinbjörnsdóttir S. (2011). Imputation of sequence variants for identification of genetic risks for Parkinson’s disease: A meta-analysis of genome-wide association studies. Lancet.

[286] Nabi R., Serajee F.J., Chugani D.C., Zhong H., Huq A.H.M.M. (2004). Association of tryptophan 2,3 dioxygenase gene polymorphism with autism. Am. J. Med. Genet..

[287] Smith A.K., Simon J.S., Gustafson E.L., Noviello S., Cubells J.F., Epstein M.P., Devlin D.J., Qiu P., Albrecht J.K., Brass C.A. (2012). Association of a polymorphism in the indoleamine-2,3-dioxygenase gene and interferon-α-induced depression in patients with chronic hepatitis C. Mol. Psychiatry.

[288] Holtze M., Saetre P., Engberg G., Schwieler L., Werge T., Andreassen O.A., Hall H., Terenius L., Agartz I., Jönsson E.G. (2012). Kynurenine 3-monooxygenase polymorphisms: Relevance for kynurenic acid synthesis in patients with schizophrenia and healthy controls. J. Psychiatry Neurosci..

[289] Golimbet V.E., Lezheiko T.V., Alfimova M.V., Abramova L.I., Kondrat’ev N.V. (2014). Association of kynurenine-3-monooxygenase gene with schizophrenia. Russ. J. Genet..

[290] Zhang S., Sakuma M., Deora G.S., Levy C.W., Klausing A., Breda C., Read K.D., Edlin C.D., Ross B.P., Wright Muelas M. (2019). A brain-permeable inhibitor of the neurodegenerative disease target kynurenine 3-monooxygenase prevents accumulation of neurotoxic metabolites. Commun. Biol..

[291] Walker A.K., Budac D.P., Bisulco S., Lee A.W., Smith R.A., Beenders B., Kelley K.W., Dantzer R. (2013). NMDA receptor blockade by ketamine abrogates lipopolysaccharide-induced depressive-like behavior in C57BL/6J mice. Neuropsychopharmacology.

[292] Kennedy P.J., Allen A.P., O’Neill A., Quigley E.M.M., Cryan J.F., Dinan T.G., Clarke G. (2015). Acute tryptophan depletion reduces kynurenine levels: Implications for treatment of impaired visuospatial memory performance in irritable bowel syndrome. Psychopharmacology.

[293] Kazemi A., Noorbala A.A., Azam K., Eskandari M.H., Djafarian K. (2019). Effect of probiotic and prebiotic vs placebo on psychological outcomes in patients with major depressive disorder: A randomized clinical trial. Clin. Nutr..

[294] Moloney G.M., O’Leary O.F., Salvo-Romero E., Desbonnet L., Shanahan F., Dinan T.G., Clarke G., Cryan J.F. (2017). Microbial regulation of hippocampal miRNA expression: Implications for transcription of kynurenine pathway enzymes. Behav. Brain Res..

[295] Valente-Silva P., Ruas J.L., Spiegelman B. (2017). Tryptophan-Kynurenine Metabolites in Exercise and Mental Health BT—Hormones, Metabolism and the Benefits of Exercise.

[296] Hardingham G.E., Bading H. (2010). Synaptic versus extrasynaptic NMDA receptor signalling: Implications for neurodegenerative disorders. Nat. Rev. Neurosci..

[297] Günther J., Däbritz J., Wirthgen E. (2019). Limitations and Off-Target Effects of Tryptophan-Related IDO Inhibitors in Cancer Treatment. Front. Immunol..

[298] Gellrich F., Schmitz M., Beissert S., Meier F. (2020). Anti-PD-1 and Novel Combinations in the Treatment of Melanoma—An Update. J. Clin. Med..

[299] Opitz C.A., Somarribas Patterson L.F., Mohapatra S.R., Dewi D.L., Sadik A., Platten M., Trump S. (2020). The therapeutic potential of targeting tryptophan catabolism in cancer. Br. J. Cancer.

